# Delivering *SaCas9* mRNA by lentivirus-like bionanoparticles for transient expression and efficient genome editing

**DOI:** 10.1093/nar/gkz093

**Published:** 2019-02-13

**Authors:** Baisong Lu, Parisa Javidi-Parsijani, Vishruti Makani, Farideh Mehraein-Ghomi, Walaa Mohamed Sarhan, Dongjun Sun, Kyung Whan Yoo, Zachary P Atala, Pin Lyu, Anthony Atala

**Affiliations:** Wake Forest Institute for Regenerative Medicine, Wake Forest University Health Sciences, Winston-Salem, NC, USA; Wake Forest Institute for Regenerative Medicine, Wake Forest University Health Sciences, Winston-Salem, NC, USA; Wake Forest Institute for Regenerative Medicine, Wake Forest University Health Sciences, Winston-Salem, NC, USA; Wake Forest Institute for Regenerative Medicine, Wake Forest University Health Sciences, Winston-Salem, NC, USA; Wake Forest Institute for Regenerative Medicine, Wake Forest University Health Sciences, Winston-Salem, NC, USA; Wake Forest Institute for Regenerative Medicine, Wake Forest University Health Sciences, Winston-Salem, NC, USA; Wake Forest Institute for Regenerative Medicine, Wake Forest University Health Sciences, Winston-Salem, NC, USA; Wake Forest Institute for Regenerative Medicine, Wake Forest University Health Sciences, Winston-Salem, NC, USA; Wake Forest Institute for Regenerative Medicine, Wake Forest University Health Sciences, Winston-Salem, NC, USA; Wake Forest Institute for Regenerative Medicine, Wake Forest University Health Sciences, Winston-Salem, NC, USA

## Abstract

The clustered regularly interspaced short palindromic repeats (CRISPR)/CRISPR-associated (Cas) system discovered using bacteria has been repurposed for genome editing in human cells. Transient expression of the editor proteins (e.g. Cas9 protein) is desirable to reduce the risk of mutagenesis from off-target activity. Using the specific interaction between bacteriophage RNA-binding proteins and their RNA aptamers, we developed a system able to package up to 100 copies of *Staphylococcus aureus Cas9* (*SaCas9*) mRNA in each lentivirus-like bionanoparticle (LVLP). The *SaCas9* LVLPs mediated transient *SaCas9* expression and achieved highly efficient genome editing in the presence of guide RNA. Lower off-target rates occurred in cells transduced with LVLPs containing *SaCas9* mRNA, compared with cells transduced with adeno-associated virus or lentivirus expressing *SaCas9*. Our LVLP system may be useful for efficiently delivering *Cas9* mRNA to cell lines and primary cells for *in vitro* and *in vivo* gene editing applications.

## INTRODUCTION

CRISPR/Cas9 (1,2) has been used as a tool to manipulate mammalian and human genomes (3–6), for gene therapy (7–9), gene expression regulation (10–12), and DNA and RNA labelling (13,14). Since its specificity is determined by the guide RNA, it distinguishes itself from zing finger nucleases and TALENS in that the same effector protein can be used for different targets. One of the major challenges of CRISPR/Cas9 technologies is the possibility of off-targets (15,16), which may cause tumorigenesis if oncogenes are hit unexpectedly. Off-target rates increase with prolonged expression of the editor proteins (17), therefore, strategies for transient delivery of the CRISPR/Cas9 components have been proposed, including conjugating Cas9 protein to cell-penetrating peptides (18), delivering Cas9 protein by electroporation (19), cationic lipid (20), and gold nanoparticles (21).

Lentiviral vector is a widely used gene delivery vehicle in research labs. It is also the gene delivery vehicle in many *ex vivo* gene therapy clinical trials (https://clinicaltrials.gov). Furthermore, lentiviral vector is also widely used for delivering the CRISPR/Cas9 machinery for efficient genome editing (17,22). Despite of this popularity, lentiviral vector mediates long term expression of the CRISPR/Cas9 machinery, which will be problematic in some situations especially in clinical applications. To achieve transient expression of genome editing proteins, various types of lentivirus-like particles (LVLPs) have been developed to deliver TALEN (23) and Cas9 protein (24), *TALEN* mRNA (25) and *Cre* mRNA (26). Using LVLP for editor protein delivery has the advantage of very transient editor protein expression (23,24). However, this strategy suffers from moderate editing efficiency and inefficient particle production. Although the latter issue can be solved by co-transfecting unmodified packaging plasmid during vector production (23,24), this need may contribute to the moderate editing efficiency due to the dilution of the editor proteins in the particles. Using LVLP for TALEN mRNA packaging through addition of the HIV packaging signal in the target mRNA is expected to package 2 mRNA molecules per particle, and the genome editing activity was unsatisfactory (25). We tried to use this strategy for transient *SaCas9* mRNA delivery and did not observe genome editing activity.

The specific interaction between RNA aptamer *MS*2 and aptamer-binding protein (ABP), *MS*2 coat protein (MCP) (27), has been used for RNA labeling (28,29) and protein recruitment (30,31). Prel *et al.* used the *MS*2/MCP pair for packaging of *Cre* mRNA in LVLPs (26). They managed to package 5–6 copies of *Cre* mRNA per particle with high Cre-mediated recombination activity (26). Considering the wide use of lentiviral vector in research and clinical applications, we decided to develop a LVLP system for transient *Cas9* mRNA delivery and efficient genome editing.

Here we describe a system to efficiently package *SaCas9* mRNA into LVLPs. The LVLPs enabled transient SaCas9 expression and highly efficient genome editing. They generated lower off-target rates compared with AAV and lentiviral delivery. Importantly, this work is not as simple as replacing *Cre* mRNA with *SaCas9* mRNA in the system described previously (26). Significant differences ensured the success of our system, which we highlight in the discussion. The *SaCas9* LVLPs described here have the transient expression feature of RNP-, mRNA- and nanoparticle-delivery strategies, but retain the transduction efficiency of lentiviral vectors. Our system may be used for packaging various editor protein-encoding mRNA for genome editing in a ‘hit-and-run’ manner.

## MATERIALS AND METHODS

### Plasmids

pRSV-Rev (Addgene #12253), pMD2.G (Addgene #12259), pMDLg/pRRE (Addgene #12251), psPAX2-D64V (Addgene #63586), pSL-MS2 × 12 (Addgene #27119), pKanCMV-mRuby3-10aa-H2B (addgene #74258) and pX601-AAV-CMV::NLS-SaCas9-NLS-3xHA-bGHpA;U6::BsaI-sgRNA (Addgene #61591) were purchased from Addgene and have been described previously. pCDH-GFP was purchased from SBI (CD513B-1). We generated the remaining plasmids (see Supplementary Table S1). Plasmids will be made available through Addgene. Gene synthesis was done by GenScript Inc. All constructs generated were sequence confirmed. Sequence information for primers, oligos and synthesized DNA fragments is in Supplementary Table S2.

### GFP reporter assay for gene editing activities

The EGFP reporter cell line described previously (32) were used to detect gene editing activity of SaCas9/human beta hemoglobin (*HBB*) sgRNA1 on the target sequences inserted in the GFP-reporter cassette. The GFP-reporter cells (derived from HEK293T cells) expressed no EGFP due to disruption of the EGFP reading frame by the insertion of the *HBB* sickle mutation and *IL2RG* target sequences between the start codon and the second codon of EGFP coding sequence. Indels formed after gene editing may restore the reading frame of the EGFP, resulting in EGFP expression. GFP-positive cells were analyzed by fluorescence microscopy or flow cytometry (BD Biosciences, Accuri C6). Single cell suspension was made in PBS/0.5% FBS for analysis. The cells without fluorescent protein expression were used as negative controls and a marker was placed at the position so that 99.9% of the cells were on the left side of the marker. In treated samples, cells on the right side of the marker were considered positive.

### AAV6 virus production and transduction

Adeno-associated virus expressing SaCas9 (33) and *HBB* sgRNA1 was made from the AAV vector pSaCas9 (expresses SaCas9) and pSaCas9-*HBB*-sgRNA1 (expresses *HBB* sgRNA1, and contains donor template for homologous recombination to change the wild-type *HBB* gene to the Sickle mutation) respectively. AAV serotype 6 (AAV6) production and concentration were performed by Virovek, Inc. (Hayward, CA, USA). For AAV6 transduction, the cells were changed to serum-free medium or OPTI-MEM, and AAV6 was added to the cells at a titer of 10^3^–10^4^ virus genome/cell. 24 h later the medium was changed to serum-containing growth medium.

### Lentivirus and LVLP production

Lentivirus was produced with the second- or the third-generation packaging systems, as described previously (32). Lentiviral vector pCK002-*HBB*-sgRNA1 expressing both *SaCas9* and *HBB* sgRNA1 was used to produce integration-competent lentivirus (packaged by packaging plasmid pspAX2 or pMDLg/pRRE), and integration-defective lentivirus (IDLV) packaged by packaging plasmid pspAX2-D64V or pMDLg/pRRE-D64V). To produce LVLPs packaged with *SaCas9* mRNA, HEK293T cells were transfected with mixed DNA of the packaging plasmid (NC-MCP or NC-PCP modified), envelope plasmid (pMD2.G), and the *SaCas9* expression plasmid with DNA ratio as shown in Supplementary Table S3. When VPR or NEF fusion proteins were used for packaging *SaCas9* mRNA, plasmid DNA for their expression was also included. Transfection was mediated by polyethylenimine (PEI, Polysciences Inc.) with a DNA: PEI ratio of 1:2. Cell culture and DNA transfection were performed as described previously (32). Twenty four hours after transfection, the medium was changed to Opti-MEM and the lentivirus or LVLPs were collected twice with one collection each 24 h. The supernatant was spun for 10 min at 500 g to remove cell debris before further processing described below.

### Concentrating lentivirus and LVLPs

Three methods were used to concentrate lentivirus and LVLPs: (i) The supernatant containing the virus or LVLPs was laid on a 10 ml 20% sucrose cushion, and then centrifuged at 20 000 g 4°C for 4 h; (ii) The supernatant containing the virus or LVLPs was mixed with the Lenti-X™ Concentrator (Takara, Cat. No. 631232) at a ratio of 3:1 (volume/volume), incubated at 4°C for 30 min and then centrifuged at 4°C 1500 × g for 45 min; (iii) the supernatant was concentrated with the KR2i TFF System (KrosFlo® Research 2i Tangential Flow Filtration System) (Spectrum Lab, Cat. No. SYR2-U20) using the concentration-diafiltration-concentration mode. Typically, 150–300 ml supernatant was first concentrated to about 50 ml, diafiltrated with 500–1000 ml PBS, and finally concentrated to ∼8 ml. The hollow fiber filter modules were made from modified polyethersulfone, with a molecular weight cut-off of 500 kDa. The flow rate and the pressure limit were 80 ml/min and 8 psi for filter module D02-E500-05-N, and 10 ml/min and 5 psi for the filter module C02-E500-05-N. Since the TFF method produced lentivirus and LVLPs with the best activities, data were generated with virus or LVLPs concentrated by the TFF system unless otherwise stated.

### Lentivirus and LVLP quantification

Viral titer was determined by p24 based ELISA (Cell Biolabs, QuickTiter™ Lentivirus Titer Kit Catalog Number VPK-107). When unpurified samples were assayed, the viral particles were precipitated according to the manufacturer's instructions so that the soluble p24 peptide was not detected.

### Transmission electron microscopy

Transmission electron microscopy was performed at the Cellular Imaging Shared Resource of Wake Forest Baptist Health Center (Winston-Salem, NC, USA). For negative staining, 30 ml virus containing supernatant was concentrated to ∼1 ml using an ultracentrifuge. Then plain carbon grids were soaked in 20 μl of virus samples and the particles were stained with phosphotungstic acid. The samples were dried and observed under a FEI Tecnai G2 30 electron microscope (FEI, Hillsboro, OR, USA).

### Western blotting analyses

To analyze viral proteins from lentivirus and LVLPs, purified lentivirus or LVLPs (200 ng p24 by ELISA) were lysed in 20 μl of 1× Laemmli sample buffer. The proteins in each sample were separated on SDS-PAGE gels and analyzed by western blotting. The antibodies used include HIV1 p17 antibody for MA (ThermoFisher Scientific, Cat. No. PA1-4954, 1:1000), HIV1 p15 polyclonal antibody for NC (Abcam, Cat. No. ab66951, 1:1000) and p24 monoclonal antibody for CA (Cell Biolabs, Cat. No. 310810, 1:1000).

To detect SaCas9 protein, HEK293T cells or GFP reporter cells were transfected with SaCas9 expression plasmid DNA or were co-transduced with 100 ng *SaCas9* mRNA packaged LVLPs and 100 ng p24 of IDLV expressing *HBB* sgRNA1. 48 hours after transfection or transduction, the cells were lysed with 100 μl of 1× Laemmli sample buffer and equal volumes of each sample was loaded for SDS-PAGE separation and Western blotting analysis by anti-HA antibody (ProteinTech, 51064-2-AP, 1:1000) and anti-Cas9 antibody (Millipore Sigma, MAB131872, clone 6F7, 1:1000) to detect the HA-tagged SaCas9. Anti-beta actin (Sigma, A5441, 1:5000) was used to compare the amount of input.

HRP conjugated anti-Mouse IgG (H+L) (ThermoFisher Scientific, Cat. No. 31430, 1:5000) and anti-Rabbit IgG (H+L) (Cat. No. 31460, 1:5000) secondary antibodies were used in Western blotting. Chemiluminescent reagents (Pierce) were used to visualize the protein signals under the LAS-3000 system (Fujifilm).

### RNA isolation from lentivirus or LVLPs

An miRNeasy Mini Kit (QIAGEN Cat. No./ID: 217004) was used to isolate RNA from concentrated lentivirus or LVLPs. Alternately, RNA was isolated directly from 140 μl particle containing supernatant with the QIAamp Viral RNA Mini Kit (QIAGEN).

### RT-PCR analysis of RNA

The QuantiTect Reverse Transcription Kit (QIAGEN) was used to reverse-transcribe the RNA to cDNA. Custom designed Hydrolysis probes specific for *SaCas9, HBB* sgRNA1 and *EGFP* (ThermoFisher Scientific) were used in qPCR, together with TaqMan Universal PCR Master Mix (ThermoFisher Scientific). PCR was run on an ABI 7500 instrument.

### Lentivirus and LVLP transduction

Various amounts of lentivirus or LVLPs (ng p24 protein) were added to 2.5 × 10^4^ cells grown in 24-well plates, with 8 μg/ml polybrene. The cells were incubated with the particle-containing medium for 12–24 h, after which normal medium was replaced.

### Gene editing in human cells

For gene editing with Cas9 expressed from AAV6, the SaCas9 expressing AAV6 and the *HBB* sgRNA1 expressing AAV6 were co-transduced into GFP reporter cells. For gene editing with LV or IDLV (packaged with packaging plasmids containing a D64V mutation in the integrase) expressing both SaCas9 and *HBB* sgRNA1, virus equivalent to 10–300 ng p24 was used to transduce 2.5 × 10^4^ cells in 24-well plates. For gene editing with LVLPs, various amounts of *SaCas9* mRNA containing LVLPs were co-transduced into HEK293T cells or the GFP reporter cells with an IDLV expressing *HBB* sgRNA1. 48–72 h after transduction, gene editing activity was analyzed by GFP-reporter assay or next-generation sequencing.

To examine gene editing in human lymphoblastoid cells immortalized by Epstein-Barr virus transformation, human lymphoblastoid cell line GM16265 was purchased from Corrie Institute. The lymphoblasts were cultured in RPMI 1640 with 2 mmol/l l-glutamine and 15% fetal bovine serum at 37°C under 5% carbone dioxide. For LVLP and IDLV transduction, 2 × 10^5^ cells were added to 0.5 ml RPMI growth medium. Then 100 ng p24 or 500 ng p24 of *SaCas9* LVLP and *IL2RG* sgRNA IDLV were added to the cells. Polybrene was added in the medium to a final concentration of 8 μg/ml. Fresh medium was replaced twenty hour hours after transduction. The cells were collected 72 h after transduction for DNA analysis by next-generation sequencing.

### Next-generation sequencing and data analyses

The following DNA regions were amplified for next-generation sequencing analysis: (i) the endogenous *HBB* target sequence, (ii) the endogenous *IL2RG* target sequence and (iii) the *HBB* target sequence in the integrated GFP-expression cassette. To amplify the endogenous *HBB* target sequence for sequencing, we used a nested PCR strategy to avoid amplifying the sequence from the viral vector template. First, primers HBB-1849F and HBB-5277R were used to amplify the 3.4 kb region from the *HBB* gene locus. These two primers cannot amplify sequences from the templates in the viral vectors. Then two inside primers HBB-MUT-F and the HBB-MUT-R primers were used to amplify the target DNA for sequencing (Supplementary Table S2; SEQ ID NOs: 34–40). To amplify the endogenous *IL2RG* target sequence, primers IL2RG-1029F and IL2RG-3301R were used to amplify the target region from the treated cells (unable to amplify sequences from the templates in the viral vectors), then two inside primers IL2RG-mut-F1 and IL2RG-mut-R4 were used to amplify the target DNA from the first PCR product for sequencing. To amplify the *HBB* target sequence from the integrated EGFP reporter for sequencing, Reporter-mut-F and Reporter-mut-R primers were used (Supplementary Table S2; SEQ ID NOs: 98–102). The proofreading HotStart® ReadyMix from KAPA Biosystems (Wilmington, MA) was used for PCR. In order to increase sequence diversity during next generation sequencing, variant numbers of stagger nucleotide were added in the 5′ primers to amplify the same target from different samples. Single-end reads from the 5′ of the PCR products were obtained by next-generation sequencing using the Illumina NextSeq 500 as described before (32). Low quality reads were removed. All sequences were sorted based on the barcodes.

After removing the 3′ linker and 5′ barcode sequences, the resulting reads were submitted to the online Cas-Analyzer software (34) for mutation analysis. When submitting the reference sequence, the primer sequences were excluded if they were at least 12 nt away from the sgRNA target. Otherwise primer sequences were included in the reference. Only those readings containing both the 5′ 12 nt and 3′ 12 nt of the reference sequence were included in Indel analysis. The total Indel rate was the difference between 1 and the percentage of readings without mutation. The top 8–10 most frequently observed readings were presented.

### *SaCas9* mRNA decay analysis

To compare the *SaCas9* RNA levels expressed from IDLV and LVLPs, 2.5 × 10^4^ HEK293 cells were transduced with 35 ng p24 of IDLV or LVLPs. IDLV particles could express SaCas9, and LVLPs were packaged with *SaCas9* mRNA. 24 h after transduction, the cells were maintained in medium containing 0.5% FBS to limit cell division. Fresh medium was changed every 48 h. The cells were collected 24, 48, 72 and 96 h post transduction, followed by RNA extraction and RT-qPCR analysis. Three hydrolysis probes, *SaCas9, GAPDH* and *RPLP0* were used for expression analysis. The relative *Cas9* mRNA levels of different particles 24 h after transduction were determined by normalization to *RPLP0*. Since no new mRNA was generated with LVLPs after transduction, no normalization was performed when evaluating mRNA decay of *Cas9* mRNA from the same particle, in order to avoid interference of possible cell proliferation on analysing RNA decay. However, *RPLP0* and *GAPDH* expression were examined for all samples to make sure sample preparation was successful and consistent.

### Statistical analysis

GraphPad Prism software (version 5.0, GraphPad Software Inc) was used for statistical analyses. T-tests were used to compare the averages of two groups. Analysis of Variance (ANOVA) was performed followed by Tukey post hoc tests to analyze data from more than two groups. Bonferroni post hoc tests were performed following ANOVA in cases of two factors. *P* < 0.05 was regarded as statistically significant.

## RESULTS

### Fusing Nucleocapsid (NC) protein with RNA-binding proteins enabled efficient packaging of *SaCas9* mRNA in lentivirus-like particles

In order to package *SaCas9* mRNA into LVLPs via specific *MS2*/MCP interaction (27), we fused *MS2*-binding protein MCP with lentiviral proteins and added MCP interacting *MS2* aptamer after the stop codon of *SaCas9* mRNA. To find the MCP fusion partner with the most efficient mRNA packaging, we explored four different lentiviral proteins, viral protein R (VPR), negative regulatory factor (NEF), nucleocapsid protein (NC) and matrix protein (MA), as the recipient proteins for MCP based on the following observation: (i) A mutant NEF (with three mutations: G3C, V153L and G177E) can be incorporated into lentiviral particles for up to 1100 molecules/capsid and has been used as a vehicle for foreign protein delivery (35,36); (ii) VPR is incorporated into the viral core for up to 550 copies/particle (37) and has also been used as the protein delivery vehicle (38); (iii) NC, which is processed from each one of the 2500–5000 Gag precursors forming a lentiviral particle (39), is the major RNA binding protein in the viral core (40); and (iv) MA, which is also processed from Gag protein, is shown to bind tRNA (41).

**Figure 1.**
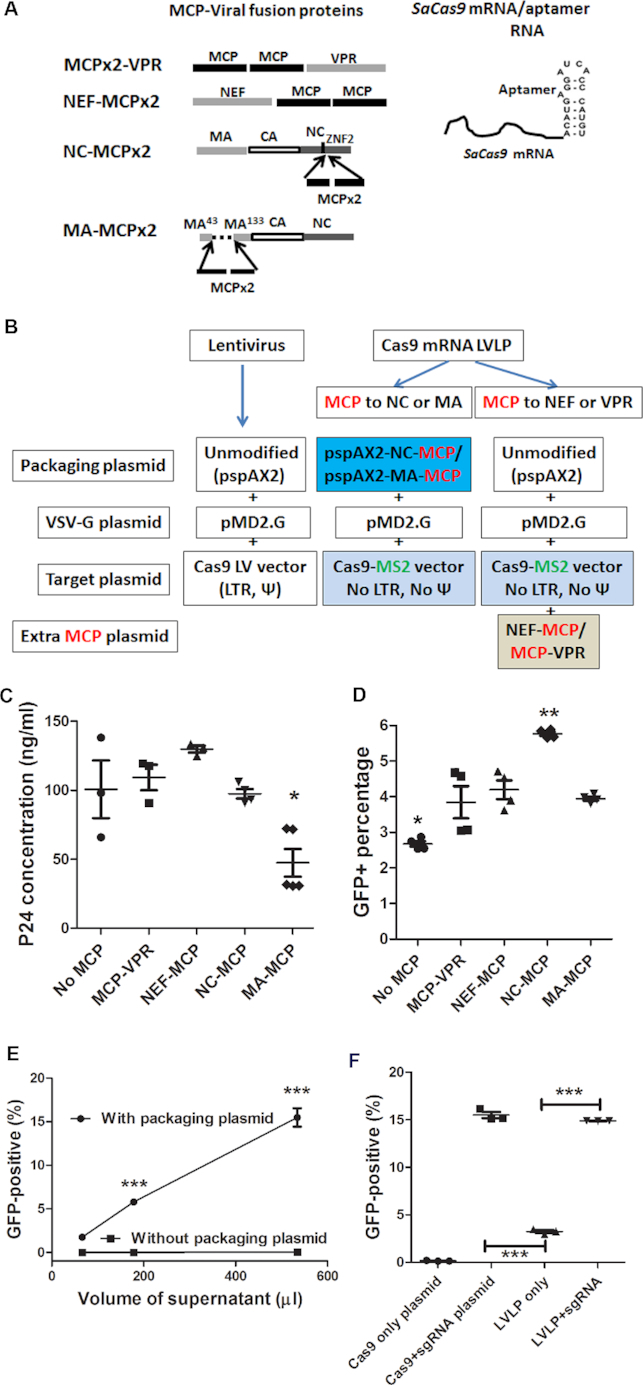
Developing LVLPs for *SaCas9* mRNA packaging. (**A**) MCP-viral protein fusion proteins and *SaCas9-MS2* aptamer fusion RNA for packaging *SaCas9* mRNA in LVLPs. MCP: *MS2*coat protein; VPR: viral protein R, NEF: negative regulatory factor; NC, nucleocapsid protein; MA: matrix protein. Dashed line indicates the deleted region in MA. (**B**) Plasmids for making lentivirus and LVLP. The aptamer-binging protein MCP and the *MS2* aptamer are shown in red and green respectively. LTR: long terminal repeats; Ψ: lentivirus packaging signal. (**C**) Effects of MCP-fusion proteins on lentivirus production. 5 × 10^5^ cells in six-well plates were transfected with the indicated plasmids (5 μg total DNA). Twenty four hours after transfection the medium was replaced with 2 ml fresh medium and p24 was assayed after 24 h. Individual data points and mean ± SEM are shown. Only MA-MCP modification decreased virus production (*P* < 0.05). (**D**) Best gene editing activity was obtained when MCP was fused to NC. *SaCas9^1xMS2^* was expressed during LVLP production. 250 μl LVLP-containing supernatant and 250 μl IDLV-expressing *HBB* sgRNA1 were co-transduced into 2.5 × 10^4^ GFP-reporter cells. ** indicates *P* < 0.01 when LVLPs from NC-MCP fusion was compared with other particles; * indicates *P* < 0.05 compared with any other particles. (**E**) Flow cytometry analysis of GFP reporter cells transduced with particles made with or without NC-MCP modified packaging plasmid. Increasing volumes of supernatants were co-transduced with 50 ng of *HBB* sgRNA1-expressing IDLV into GFP reporter cells. When producing particles without packaging plasmid, the packaging plasmid was replaced with pKanCMV-mRuby3-10aa-H2B expressing mRuby. ***P < 0.001 when the GFP-positive rates of the two conditions were compared (Bonferroni posttests following ANOVA). (**F**) Plasmid DNA contributed little in generating GFP-positive reporter cells. pSaCas9^1xMS2^ (first column) or pSaCas9^1xMS2^-*HBB* sgRNA1 plasmid DNA (second column) was transfected into GFP reporter cells to observe GFP-positive cells. pSaCas9^1xMS2^-*HBB* sgRNA1 was also used to make LVLPs to transduce into GFP reporter cells alone (100 ng p24, the third column) or with 50 ng p24 of IDLV expressing *HBB* sgRNA1 (the fourth column). *** indicates *P* < 0.001. For C, D and F, Tukey's multiple comparison tests were performed following ANOVA analysis.

We thus fused MCP with NEF and VPR in the configuration shown in Figure 1A as reported previously (35,36,38). MCP-VPR and NEF-MCP were expressed from co-transfected plasmid DNA during LVLP production with the unmodified packaging plasmid (Figure 1B). To fuse MCP with NC, we modified the packaging plasmid by inserting MCP coding sequence after that of the second zinc finger of NC. Because the second zinc finger of NC is necessary for virion production and deleting this domain reduced virion production 10-fold (42), we chose to preserve this domain rather than replacing it with MCP as reported previously (26). To fuse MCP with MA, we replaced the coding sequence for MA amino acid 44–132 in the packaging plasmid with that of MCP, since this region of MA is not necessary for virus production (43). We inserted MCP coding sequence into NC or MA of the packaging plasmid in the way that the Gag precursor protein reading frame and the protease processing sites were preserved.

To examine whether the incorporation of MCP affected virion production, we compared the GFP-lentivirus production efficiency using these constructs. Expressing MCP-VPR or NEF-MCP in packaging cells did not affect GFP lentivirus production with the original packaging plasmid. NC-MCP modified packaging plasmid generated GFP lentivirus with similar efficiency as the original packaging plasmid, but MA-MCP modified packaging plasmid showed reduced virus production efficiency (Figure 1C, Supplementary Excel File 1, sheet 1).

In order to have a simple assay to compare genome editing activities, we used the GFP-reporter cells (derived from HEK293T cells transduced with GFP-reporter lentiviral vectors) described recently (32). These GFP-reporter cells were integrated with a GFP expression cassette, wherein the Sickle mutation sequence of human beta hemoglobin (*HBB*) targeted by *HBB* sgRNA1 was inserted between the *EGFP* start and the second codons, disrupting the reading frame. Insertions or deletions (Indels) in the target sequence resulting from gene editing may restore the *EGFP* reading frame and GFP expression. To test whether fusing MCP to virus proteins could enhance the packaging of *SaCas9* mRNA into LVLPs, *SaCas9^1xMS2^* (*SaCas9* mRNA with 1 copy of *MS2* aptamer after the stop codon) was expressed during LVLP generation by the original packaging plasmid (with or without MCP-VPR or NEF-MCP expression), or by the MCP modified packaging plasmid (Figure 1B). These LVLPs were then co-transduced into the GFP-reporter cells with an integration-defective lentiviral (IDLV) vector expressing *HBB* sgRNA1 targeting the Sickle mutation of human beta hemoglobin (*HBB*) (32). LVLPs made from NC-MCP modified packaging plasmid generated significantly more GFP-positive reporter cells than LVLPs generated by all other strategies of MCP incorporation (*P* < 0.01) (Figure 1D, Supplementary Excel File 1, sheet 2). NC was thus used as the MCP recipient protein for further experiments.

MCP dimers, but not the monomers or oligomers, had RNA aptamer-binding activities (44). When we compared the performance of packaging plasmids wherein NC was fused with one or two copies of MCP, LVLPs generated by packaging plasmid fused with one copy of MCP produced more GFP^+^ reporter cells than that fused with two copies of MCP (5.7 ± 0.1%, *N* = 4, versus 4.4% ± 0.3%, *N* = 4; *P* < 0.01. Supplementary Excel File 1, sheet 3). Thus, the modified packaging plasmid fused with one copy of MCP was used in subsequent experiments.

To test whether the gene editing activity observed was caused by nucleic acid in extracellular vesicles promoted by VSV-G (45), we included a control where the NC-MCP modified packaging plasmid was replaced with the plasmid expressing mRuby fluorescent protein but none of the lentivirus packaging proteins. Equal volumes of supernatants were co-transduced onto GFP-reporter cells with *HBB* sgRNA1 expressing IDLV, supernatant produced with the packaging plasmid generated up 15% GFP^+^ reporter cells. Whereas supernatant produced without packaging plasmid generated lower than 0.5% GFP^+^ reporter cells under all conditions (Figure 1E, Supplementary Excel File 1, sheet 4); and the mRuby positive rate was lower than 0.5% in cells treated with the highest volume of supernatant (Supplementary Figure S1). Since VSV-G was present in both conditions, the data suggested that extracellular vesicles did not have a major role in generating GFP^+^ reporter cells. The data also suggested that residual plasmid DNA contributed little in the total gene editing activities observed.

To further test the contribution of residual plasmid DNA from LVLP production in generating GFP^+^ reporter cells, constructs expressing only *SaCas9^1xMS2^*mRNA (pSaCas9^1xMS2^) and expressing both *SaCas9^1xMS2^*mRNA and *HBB* sgRNA1 (pSaCas9^1xMS2^-*HBB* sgRNA1) were made. As expected, transfecting pSaCas9^1x^*^MS^*^2^-*HBB* sgRNA1 plasmid DNA into the GFP reporter cells generated significantly more GFP^+^ reporter cells than transfecting pSaCas9^1x^*^MS^*^2^ plasmid DNA (Figure 1F). Since *HBB* sgRNA1 without the *MS2* aptamer could not be packaged, LVLPs made from pSaCas9^1xMS2^-*HBB* sgRNA1 plasmid DNA were expected to contain insufficient copies of *HBB* sgRNA1. Indeed, these LVLPs produced significantly less GFP^+^ reporter cells when transduced alone than when co-transduced with IDLV expressing *HBB* sgRNA1 (*P* < 0.0001, Figure 1F, Supplementary Excel File 1, sheet 5). Note that in this experiment, nonspecifically packaged sgRNA in LVLPs also contributed to the background activity, which explains why higher background activity was observed in Figure 1F than in Figure 1E. These observations argue against a major contribution from plasmid DNA left over during LVLP production, and support more effective packaging of SaCas9^1xMS2^ mRNA when using NC-MCP LVLPs.

### Human beta hemoglobin (*HBB*) 3′ untranslated region greatly improved the genome editing activities of *SaCas9* mRNA packaged in LVLPs

**Figure 2.**
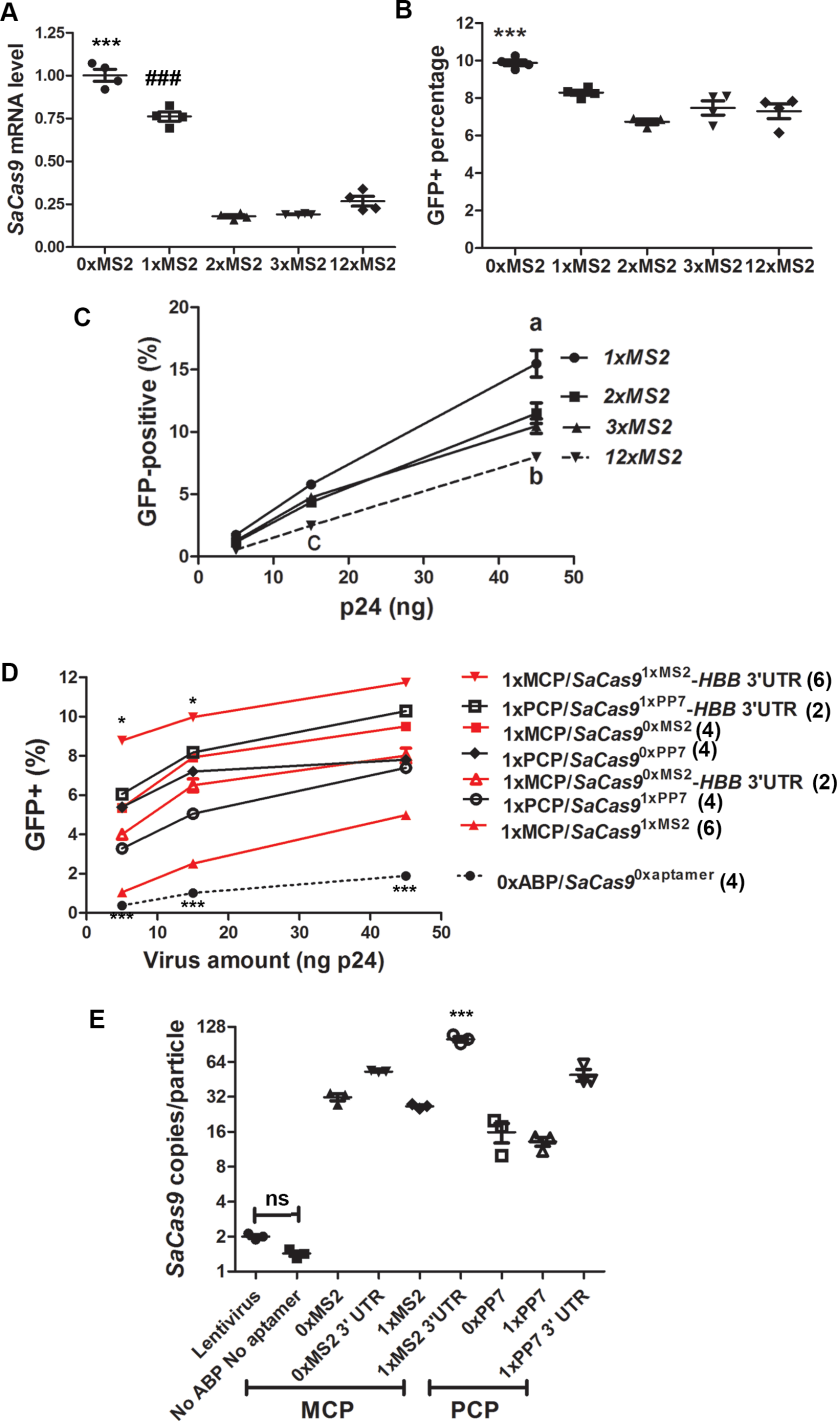
Gene editing activities of various LVLPs. (**A**) Effects of *MS2* aptamers on *SaCas9* mRNA level. Plasmid DNA expressing *SaCas9^nxMS2^* (250 ng) was co-transfected into GFP-reporter cells grown in 24-well plates with plasmid DNA expressing *HBB* sgRNA1 (250 ng). *SaCas9* mRNA level was compared by RT-qPCR with *HBB* sgRNA1 as a control. *** and ### indicate *P* < 0.001 when *SaCas9^0xMS2^* was compared with *SaCas9^1xMS2^*, and when *SaCas9^1xMS2^* was compared with *SaCas9* with more than one *MS2* aptamer. (**B**) Effects of *MS2* aptamers on *SaCas9* gene editing activity. GFP-reporter cells were transfected as in A. Flow cytometry was performed 72 h after transfection. *** indicates *P* < 0.001 when *SaCas9^0xMS2^* was compared with *SaCas9* with at least one *MS2* aptamer. (**C**) Examining the effects of aptamer numbers on LVLP gene editing activity. LVLP-containing supernatants (titer determined by p24 ELISA) were co-transduced with 50 ng *HBB* sgRNA1-expressing IDLV into 2.5 × 10^4^ GFP-reporter cells. Flow cytometry was performed 72 h after transduction. ‘a’ indicates that 45 ng p24 of *SaCas9^1xMS2^* LVLPs obtained significantly higher GFP-positive rate (*P* < 0.001) than all other LVLPs at the same dosage; ‘b’ indicates that 45 ng p24 of *SaCas9^12xMS2^* LVLPs obtained significantly lower GFP-positive rate (*P* < 0.001) than *SaCas9^2xMS2^* or *SaCas9^3xMS2^*LVLPs; ‘c’ indicates that 15 ng p24 of *SaCas9^12xMS2^* LVLPs obtained significantly lower GFP-positive rate (*P* < 0.05) than all other LVLPs at the same dosage. Each data point is the mean of three replicates. (**D**) Flow cytometry analysis of GFP-positive cells generated after transducing various *SaCas9*-containing LVLPs. Designated amounts of *SaCas9*-containing LVLPs were co-transduced with 60 ng p24 *HBB* sgRNA1-expressing IDLV into 2.5 × 10^4^ GFP-reporter cells. Each point was the average of indicated replicates (numbers in parentheses). * indicates *P* < 0.05 when *SaCas9*^1x^*^MS^*^2^-*HBB* 3′ UTR LVLP was compared with any other particles of the same dosage; *** indicates *P* < 0.001 when LVLPs generated without ABP and aptamers were compared with any other particles of the same dosage. (**E**) RT-qPCR comparisons of *SaCas9* mRNA copy numbers in different types of LVLPs. RNA was purified from lentivirus or LVLPs containing 30 ng p24. 30 ng p24 of GFP-lentivirus was added in each sample for experimental control. Copy numbers were compared to normal lentiviral vectors known to contain two RNA genomes/LV particle. Shown are individual data points and mean ± SEM. ns, no significant difference; ***P < 0.001 compared with any other groups. For A, B and E, Tukey's multiple comparison tests were performed following ANOVA analysis. For C and D, Bonferroni posttests were performed following two-way ANOVA.

To determine the best copy number of *MS2* aptamer for packaging of *SaCas9* mRNA, we first examined the effects of 0, 1, 2, 3 and 12 copies of *MS2* aptamers on *SaCas9* mRNA expression. Equal amounts of plasmid DNA expressing *SaCas9^nxMS2^* (*n* indicates 0, 1, 2, 3 or 12) was transfected into HEK293T cells and the steady-state level of *SaCas9* mRNA was compared by RT-qPCR. Addition of 1 aptamer after the stop codon of *SaCas9* slightly decreased the steady-state level of *SaCas9* mRNA, while adding more than 1 aptamer significantly decreased *SaCas9* mRNA (*P* < 0.001, Figure 2A, Supplementary Excel File 1, sheet 6). Consistent with the decrease in mRNA, when *SaCas9^nxMS2^*-expressing DNA was co-transfected into our GFP reporter cells with the plasmid expressing the *HBB* sgRNA1, one aptamer slightly decreased the percentage of GFP^+^ reporter cells, while more aptamers caused a further decrease (Figure 2B, Supplementary Excel File 1, sheet 7). Consistently, when LVLPs containing *SaCas9^1xMS2^, SaCas9^2xMS2^, SaCas9^3xMS2^*, or *SaCas9^12xMS2^* were co-transduced into GFP-reporter cells with *HBB* sgRNA1-expressing lentivirus, *SaCas9^1xMS2^* LVLPs generated the most GFP-positive cells, and *SaCas9^12xMS2^* LVLPs generated the least at all concentrations tested (Figure 2C, Supplementary Excel File 1, sheet 8).

To enhance *SaCas9* mRNA stability and translatability with or without aptamer, we added two copies of 3′ untranslated region (UTR) sequences from the human *HBB* gene (46,47) after the *SaCas9* stop codon but before the *MS2* aptamer. Plasmid DNA transfection in HEK293T cells showed that the steady-state level of *SaCas9*^1xMS2^-*HBB 3*′ *UTR* was 1.3-fold of that of *SaCas9*^0xMS2^ (1.3 ± 0.08 versus 1.0 ± 0.03, *n* = 4, *P* < 0.05, Supplementary Excel File 1, sheet 9).

Since more than one *MS2* aptamer significantly decreased the gene editing activities of the LVLPs, one copy of *MS2* aptamer was further studied. We made LVLPs containing *SaCas9* mRNA with or without *MS2* aptamer and *HBB* 3′UTR (*SaCas9^0xMS2^, SaCas9^1xMS2^, SaCas9^0xMS2^-HBB 3*′*UTR, SaCas9^1xMS2^-HBB 3*′*UTR*), and transduced each type of particles into the GFP-reporter cells with *HBB* sgRNA1-expressing IDLV. Flow cytometry analysis revealed that without MCP, the LVLP produced GFP-positive cells inefficiently (dashed lines in Figure 2D, Supplementary Excel File 1, sheet 10). Among LVLPs generated with MCP, the presence of both *MS2* and *HBB* 3′UTR resulted in the highest genome editing activity. Surprisingly, *SaCas9* mRNA without *MS*2 aptamer can also generated appreciable GFP-positive cells. *SaCas9^1xMS2^* LVLPs showed lower activities than *SaCas9^0xMS2^* LVLPs, consistent with our previous observation of *MS2* decreasing *SaCas9* mRNA stability. *HBB* 3′UTR significantly improved the performance of *SaCas9^1xMS2^* LVLPs but not that of *SaCas9^0xMS2^* LVLPs, another observation consistent with our previous data. Our data suggest that (i) MCP is necessary for efficient packaging of *SaCas9* mRNA; (ii) *MS2* aptamer decreases *SaCas9* mRNA stability, although they may increase *SaCas9* mRNA/NC-MCP association and (iii) *HBB 3*′*UTR* increased *SaCa9* mRNA stability, and the presence of *MS2* and *HBB 3*′*UTR* improved *SaCas9* mRNA/NC-MCP association and *SaCa9* mRNA stability.

Considering the possible packaging of dCas9 mRNA for CRISPR-mediated gene regulation, where sgRNA may need to be modified with aptamers to enhance gene regulation, more than one aptamers may be used in the same experiment. We also developed a *PP7/PP7*coat protein (PCP) based packaging system to enable aptamer combinations. *PP7*/PCP is another RNA aptamer/ABP pair used in RNA studies (29,48). We replaced MCP by PCP in the packaging plasmid and replaced the *MS2* aptamer by the *PP7* aptamer in the *SaCas9* mRNA expression plasmid (expressing *SaCas9^1xPP7^*). *PP7*/PCP packaged *SaCas9* LVLPs could also efficiently generate GFP^+^ reporter cells when co-transduced into the GFP-reporter cells with the *HBB* sgRNA1-expressing IDLV (Figure 2D, Supplementary Excel File 1, sheet 10). Surprisingly, PCP-modified packaging system can also package *PP7*-free *SaCas9* mRNA, and in this case, the packaging is PCP-dependent.

The GFP-reporter assay data were corroborated by RT-qPCR analysis of *SaCas9* mRNA copy numbers per LVLP (Figure 2E). Consistent with the observation that LVLPs containing *SaCas9^1xMS2^-HBB 3*′*UTR* resulted in the highest percentage of GFP-positive cells, they had the highest *SaCas9* mRNA copy number (∼50-fold RNA molecules contained in lentiviral vectors with similar p24 protein). Although *SaCas9^0xMS2^* and *SaCas9^1xMS2^* LVLPs had similar copies of *Cas9* mRNA/particle, the gene editing activity of *Cas9^1xMS2^* LVLPs was consistently lower than that of *Cas9^0xMS2^* LVLPs (Figure 2D). This could be the result of the *MS2* aptamer decreasing *SaCas9* mRNA stability.

Comparing the LVLPs packaged by MCP/*MS2* and PCP/*PP7* based systems, PCP/*PP7* packaged *SaCas9* LVLPs had better gene editing activities than those of MCP/*MS2* packaged LVLPs when *HBB* 3′UTR was not added to *SaCas9* mRNA. The opposite was true when *HBB* 3′UTR was added to *SaCas9* mRNA (Figure 2D). *SaCas9^1xMS2^-HBB* 3′UTR LVLPs consistently showed the best gene editing activity in multiple repeats (Supplementary Excel File 1, sheet 10).

**Figure 3.**
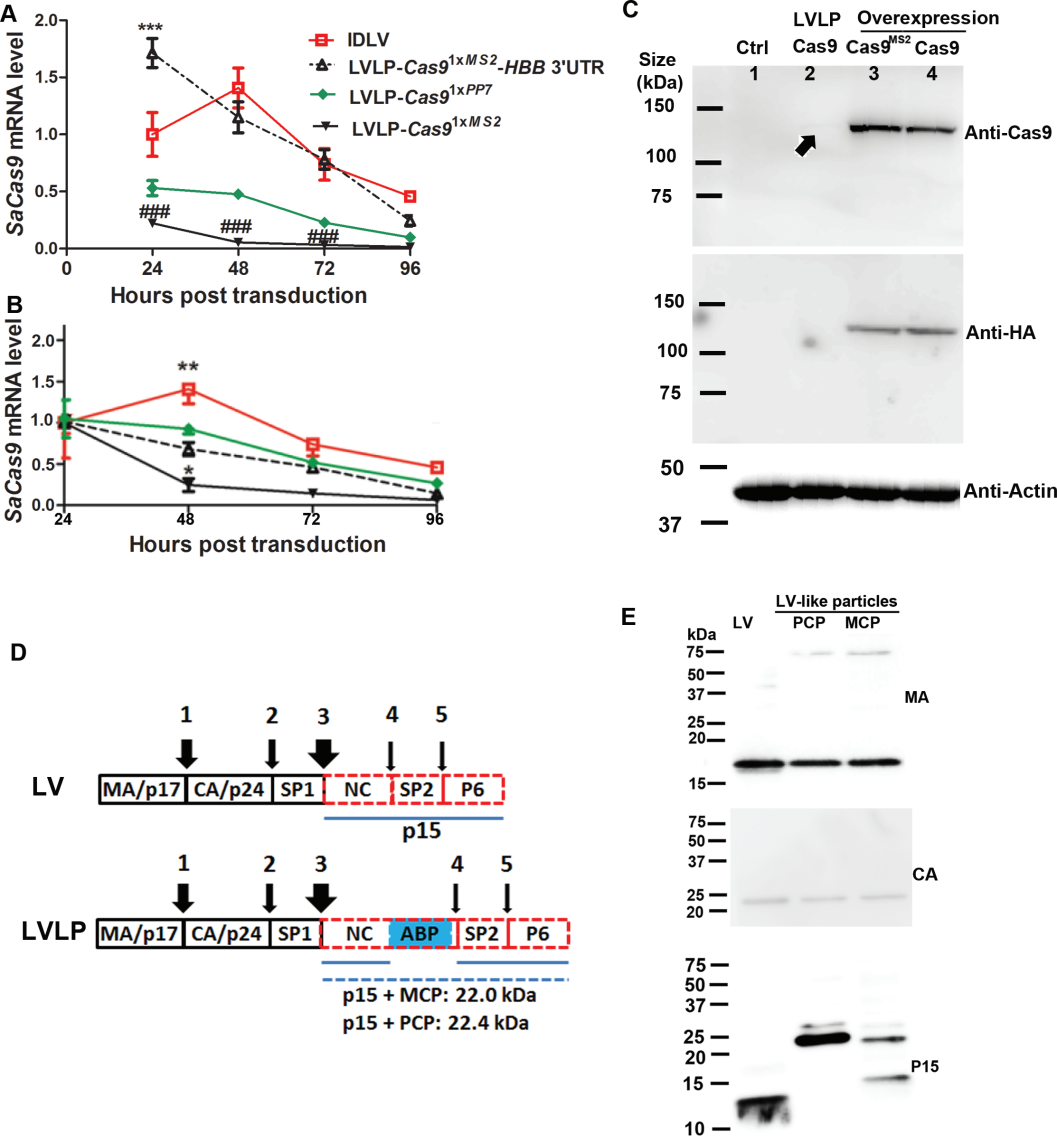
Characterization of LVLPs. (**A**) Transient expression of *SaCas9* mRNA from LVLPs. *SaCas9-*expressing IDLV (35 ng p24) or LVLPs (35 ng p24) were transduced into 2.5 × 10^4^ HEK293T cells. *SaCas9* mRNA levels were assayed at different time points. *SaCas9* mRNA levels 24h post transduction were normalized by housekeeping gene *GAPDH* and *RPLP0* to obtain the relative *SaCas9* mRNA expression level. No normalization was performed thereafter so that possible cell replication does not affect evaluation of mRNA degradation since no new mRNA was generated in LVLP transduced cells. *** indicates *P* < 0.001 when *SaCas9*^1x^*^MS^*^2^-*HBB* 3′UTR was compared with other particles at the same time. ### indicates *P* < 0.001 when *SaCas9*^1x^*^MS^*^2^ was compared with other particles at the same time. (**B**) Time course of *SaCas9* mRNA level from the same particle. mRNA levels of each particle 24h post transduction were set as 1. Shown are mean ± SEM of indicated replicates. * and ** indicate *P* < 0.05 and *P* < 0.01 when compared with other particles at the same time point. For A and B, Bonferroni posttests were performed following two-way ANOVA. (**C**) Western blotting analysis of SaCas9 protein. The four lanes were lysates from mock transfected HREK293T cells (lane 1), GFP-reporter cells co-transduced with 300 ng p24 of *Cas9* LVLP and 50 ng p24 of *HBB* sgRNA1 IDLV (lane 2), HEK293T cells overexpressing *Cas9*^1x^*^MS2^* (lane 3) or *Cas9* mRNA (lane 4) by transfecting 0.25 μg DNA to 1.25 × 10^5^ cells. A very faint band in LVLP transduced cells was indicated by an arrow. (**D**) Diagram showing the processing of Gag precursor by HIV protease. The wideness of the arrows is proportional to the processing speed at that site (1–5). The estimated sizes of the p15-ABP fusion proteins were listed. (**E**) Western blotting analysis of lentiviral proteins. 200 ng p24 of GFP lentivirus, NC-MCP and NC-PCP modified LVLPs were analyzed.

We examined the expression duration of the mRNAs delivered by the LVLPs and compared them with that of IDLV. Twenty four hours after transduction, *SaCas9^1xMS2^-HBB* 3′UTR LVLPs showed the highest *Cas9* mRNA level, consistent with their high mRNA copy number/particle and mRNA stability (Figure 3A, Supplementary Excel File 1, sheet 11). *SaCas9^1xMS2^* LVLPs had the lowest mRNA expression, consistent with their low gene editing activity. In contrast to IDLV’s increased *Cas9* mRNA expression at 48 h post transduction, suggesting transcription of new *Cas9* mRNA, all mRNAs delivered by LVLPs decreased steadily with different speed. Compared with the respective mRNA levels of 24 h post transduction, *SaCas9^1xPP7^* showed similar decay rates as *SaCas9^1xMS2^-HBB 3*′*UTR* (Figure 3B, Supplementary Excel File 1, sheet 12), while *SaCas9^1xMS2^* showed much faster decay rates. This could also explain why *SaCas9^1xMS2^-HBB* 3′UTR and *SaCas9^1xPP7^* performed better than *SaCas9^1xMS2^* in GFP-reporter assays. Thus, *SaCas9^1xMS2^-HBB* 3′UTR LVLPs were able to achieve transient and high expression compared to IDLV viral vectors.

We attempted to examine the protein expression from the LVLPs. We could readily detect Cas9 protein expression in overexpressed HEK293T cells or GFP reporter cells with either the SaCas9 or HA (the Tag at the C-terminus of Cas9) antibodies, but could hardly detect Cas9 expression in GFP-reporter cells 48 h after transducing even 300 ng LVLPs (Figure 3C). These cells were co-transduced with *HBB* sgRNA1 IDLV and 15% of them were GFP-positive in flow cytometry analysis, suggesting successful gene editing in ∼45% of the cells, even though Cas9 protein were difficult to detect in these cells by Western blotting. The data showed that the LVLPs were able to express sufficient Cas9 protein for efficient gene editing, although hardly detectable by the method we used. Since the rate of off-targets increases with increasing Cas9 protein (49), the relative low Cas9 protein from LVLPs will also ensure low off-targets.

The Gag precursor protein was processed into mature proteins by sequential protease cleavage with different cleavage speed at different site (50) (Figure 3D). We used antibodies specific for MA (p17), CA (p24) and p15 (from which NC is processed) to analyze the sizes of the processed proteins in normal GFP lentiviral vectors, MCP- based *SaCas9^1xMS2^* LVLPs, and PCP-based *SaCas9^1xPP7^*LVLPs. MA and CA protein from all particles showed the same expected size, indicating proper cleavage at the time of analysis (Figure 3E). Anti-MA antibody detected weak 75 kDa bands in MCP and PCP-based LVLPs. However, similar bands were not detected by anti-CA or anti-p15 antibodies, arguing against the presence of unprocessed Gag precursor proteins. The anti-p15 antibody detected a band slightly smaller than 15 kDa in GFP lentivirus, similar to earlier observation (51). This size indicates that at the time of analysis, cleavage at sites 4 and 5 did not occur to the detectable level, consistent with their low speed (50). p15 antibody detected bands between 20–25 kDa in PCP- and MCP-based LVLPs, matching the expected sizes of NC-PCP and NC-MCP fusion proteins in LVLPs (Figure 3D). In MCP-based LVLPs, an additional small band was detected by the p15 antibody, indicating partial degradation of the p15-MCP fusion protein. These data suggest that the insertion of MCP or PCP in NC had little effect on Gag precursor cleavage at sites 1 (MA/CA), 2 (CA/SP1) and 3 (SP1/NC). The effects on cleavage at sites 4 (NC/SP2) and 5 (SP2/P6) were unknown since at the time of analysis these sites were not cleaved even in normal LV, judged from the size of peptide detected by p15 antibody. Electron microscopy analyses of the MCP- and PCP-based lentiviral-like particles revealed similar particle sizes as GFP lentivirus (Supplementary Figure S2).

### LVLP packaged *SaCas9* mRNA enables highly efficient genome editing

**Figure 4.**
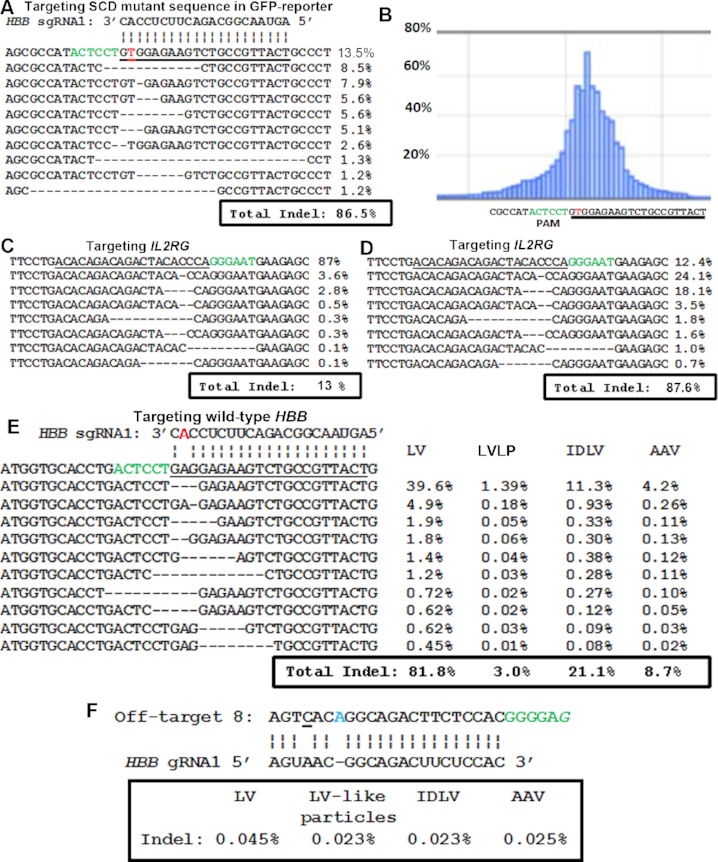
Genome editing by *SaCas9* LVLP. (**A**) *SaCas9* LVLPs efficiently generated Indels in the perfectly matched *HBB* sgRNA1 target sequence. The most frequently observed sequences and their percentages from next-generation sequencing are listed. The ‘T’ in red is the mutation in *HBB* causing Sickle cell disease. (**B**) Frequency of Indels at each position obtained from sequencing data in A. (**C**) Indels in *IL2RG* gene of HEK293T cells generated by *SaCas9* LVLPs. 2.5 × 10^4^ cells were co-transduced with 30 ng p24 of *SaCas9^1xMS2^-HBB* 3′UTR LVLPs and 60 ng of IDLV expressing *IL2RG* sgRNA. (**D**) Indels in *IL2RG* gene of lymphoblasts generated by 500 ng p24 of *SaCas9^1xMS2^-HBB* 3′UTR LVLPs (500 ng p24 LVLPs on 2 × 10^5^ cells). (**E**) Indel rates in the wild-type *HBB* locus of the GFP reporter cells. There was one mismatch between the *HBB* sgRNA1 (highlighted in red) and the target sequence. (**F**) Indel rates in predicted off-target 8 of the GFP reporter cells. Off-target 8 has one mismatch with *HBB* sgRNA1 (underlined), one DNA bulge (in blue) and a non-typical PAM (a ‘G’ instead of a ‘T’ at the last position, in italics). For A–F: The protospacer adjacent motif (PAM) or its complementary sequence is in green. For A–E: The target sequences are underlined. Vertical lines indicate complementarity between *HBB* sgRNA1 and the target. 3–4 million readings were obtained for each sample.

To further determine the gene editing activity of the *SaCas9* mRNA packaged LVLPs, we co-transduced LVLPs (30 ng p24 protein) packaged with *SaCas9*^1xMS2^-*HBB* 3′UTR mRNA into 2.5 × 10^4^ GFP reporter cells with 60 ng p24 of *HBB* sgRNA1-expressing IDLV. 48 hours after transduction, 13% of the reporter cells became GFP-positive. The target DNA for *HBB* sgRNA1 in the GFP expression cassette was amplified and sequenced by next-generation sequencing. Overall, 86.5% of the alleles had Indels (Figure 4A), mostly around the predicted cleavage site, which is 3 nt away from the PAM (Figure 4B). The data demonstrated that delivering *SaCas9* mRNA by the LVLPs is highly efficient in generating Indels.

To test the activities of *SaCas9* LVLPs on endogenous targets, we prepared IDVL expressing sgRNA targeting human *IL2RG*, mutation of which causes X-linked severe combined immunodeficiency (SCID-X1). In HEK293T cells, 30 ng p24 of *SaCas9* LVLPs and 60 ng of *IL2RG* sgRNA IDLV generated 13% Indels in the target sequence (Figure 4C). In human lymphoblasts (2 × 10^5^ cells), co-transduction of 100 or 500 ng p24 of both types of particles generated 11% or 87.6% Indels in the target sequence (Figure 4D). Although the Indel rates were different under different conditions, the top most frequently observed mutations were the same. The data showed that the *SaCas9* LVLPs could be used to target different target genes in multiple cells.

### LVLP packaged *SaCas9* mRNA showed lower off-target rates than those of viral vectors

The *HBB* sgRNA1 was designed to target the *HBB* Sickle cell mutation and has one nucleotide mismatch with the wild-type *HBB* sequence (Figure 4E). As such, the corresponding wild-type *HBB* gene sequence in the GFP reporter cells can be regarded as the ‘off-target’ for SaCas9/*HBB* sgRNA1. Indel rates in the wild-type *HBB* locus were compared when SaCas9 was delivered by LVLP, AAV6, IDLV, or LV expressing SaCas9. To control the percentage of cells having functional SaCas9/*HBB* sgRNA1, GFP-positive cells were sorted by GFP-activated sorting 48 hours after transduction. At the time of sorting (48 hours after transduction), the percentage of GFP-positive rate was 11.1%, 2.1%, 8.5% and 6.5% for cells treated with LV, LVLP, IDLV, and AAV6 (10^4^ vg/cell), respectively. Although we treated the cells with the same number of LV, LVLP and IDLV particles based on p24 amount (about 750 ng p24 for 1.25 × 10^6^ cells), integration-competent LV treated cells already showed higher GFP-positive rates than IDLV due to continuous CRISPR/Cas9 expression. After sorting, the GFP-positive rates were 95.4%, 88.9%, 93.3% and 90.8% respectively, suggesting the presence of functional SaCas9/*HBB* sgRNA1 in most cells under each condition. The DNA from the endogenous *HBB* locus was then amplified from the sorted cells 1 week after sorting and subjected for next-generation sequencing. The LVLP system had the lowest Indel rate in the endogenous *HBB* site and LV had the highest (Figure 4E). Our data demonstrate that delivering *SaCas9* mRNA by LVLPs for gene editing is safer than other viral delivery systems.

In the cells described above, we also searched for Indels in 9 possible off-targets predicted by Cas-Offinder (52) and CRISPOR (53), we failed to detect Indels in 8 of the 9 potential *HBB* sgRNA1 off-targets even in LV treated cells (unpublished data). However, in off-target 8 (chromosome 20, 33982230bp-33982337bp), we detected slightly more Indels in LV-transduced cells than in LVLP transduced cells (Figure 4F). Due to the relative low off-target rates under both conditions, the significance of the difference needs more analysis. Our observation of low off-target rates from SaCas9 is consistent with previous reports (54,55). Ours and previous observations that transient Cas9 action decreases off-target rates (17,19) suggest that delivering SaCas9 by LVLPs will improve safety.

Since we have GFP-reporter cells transduced with different particles that showed different percentages of GFP-positive cells (cells used in Figure 4A and unsorted cells for Figure 4E), we analyzed the relationship between GFP-positive percentages and target sequence (in the GFP-expression cassette) Indel rates in these cells. We found that the Indel rates increased with the GFP-positive percentage linearly (Supplementary Figure S3, Supplementary Excel File 1, sheet 13, *r*^2^ = 0.9497 by linear regression). The data validated our GFP-reporter assay for comparing the genome editing activities of different particle types.

## DISCUSSION

Here we describe *SaCas9* mRNA LVLPs for transient Cas9 expression and highly efficient genome editing. Although other types of LVLPs have been described for delivering proteins or mRNAs (23–26), the LVLP we developed has unique features which will make it a useful tool in the expanding genome editing toolbox. Since we found that using the HIV packaging signal to package mRNA in LVLP as described previously (25) did not work with *SaCas9* mRNA (our unpublished observation), we will compare our system with the other three types of LVLPs (23,24,26), by comparing their packaging and delivery efficiency, and then by comparing their Cas9 activity.

The LVLP described here can be efficiently produced without the need to include unmodified packaging plasmids, which is a requirement in the protein delivery of previous LVLP systems (23,24). With our LVLPs system, we observed similar efficiencies making LVLP and standard LV. Whereas when making LVLPs for Cas9 protein delivery, the particle production efficiency was 3–5-fold lower compared to standard LV even when the original packaging plasmid was included (24). The RNA delivery LVLP system described by Prel *et al* (2015) did not compare their LVLP production efficiency with that of standard LV (26). However, they could only obtain up to 27.4 μg/ml p24 after 1000-fold concentration, indicating 27.4 ng/ml before concentration. This yield is much lower than our typical yield of 100 ng/ml, when particles produced during 24 and 48 hours after transfection were assayed. Their low particle yield is most likely the result of removing zinc finger 2 of NC, since it was reported earlier that doing so will decrease lentivirus production by over 10 fold (42). In addition to low particle yield, the Gag precursor proteins are not efficiently processed in their LVLPs. While in our LVLPs, the Gag precursor processing is essentially unaffected and similar to that of standard LV, which could also contribute to the high packaging efficiency and activity of the LVLPs described in this study.

Our *Cas9* mRNA LVLP is highly active in genome editing. In HEK293T cells and lymphoblasts, we observed >85% Indel rates in different targets with 1.2–2.5 pg p24 LVLP/cell. The ZNF and TALEN protein delivering LVLPs generated up to 24% Indel rates with 2∼6 pg p24 LVLP/cell (23), and the Cas9 protein delivering LVLP observed up to 19.5% Indel rates with 0.75 pg p24 LVLP/cell (24). Prel *et al.* focused on using their RNA delivering LVLPs to package mRNAs other than *Cas9* mRNA (26). However, they reported in supplementary data that their *Cas9* mRNA LVLPs (20 pg p24/cell) could disrupt GFP expression in ∼25% reporter cells with two gRNAs (26). Theoretically about two-thirds of the Indels will disrupt reading frame, which indicates that their Indel rate could be ∼37.5%. Apparently our LVLPs have the best genome editing activity compared with all reported LVLPs.

The following factors contributed to the high genome editing activity of our *Cas9* mRNA LVLPs compared to protein delivery LVLPs: 1) the Cas9 protein expressed from our LVLPs is the same as the Cas9 protein expressed from commonly used viral vectors and plasmid DNA, while the editor proteins from the protein delivering LVLPs have to fuse to the N-termini of Gag (23,24). Not only the folding and function of the editor proteins may be compromised, the editor proteins are also subjected to extensive HIV protease degradation (23,24). In addition, the need to include original packaging plasmid to enhance viral particle production (24) also contributed to the low activity due to the decrease of the editor proteins that can be incorporated per particle.

Compared to the RNA delivery LVLPs described by Prel *et al.* (26), we used one aptamer while they added 12 *MS2* aptamers to the mRNA to be packaged. Multiple copies of aptamers are necessary for RNA labelling experiments since they can enhance the signal from the labelled RNAs and lower the background from the unbound GFP-MCP fusion protein (56). However, for the purpose of RNA packaging, multiple aptamer copies may not be a good choice. On one hand, multiple aptamer copies may decrease the stability of RNAs to be packaged. We observed that more than one copy of aptamer decreased *SaCas9* mRNA stability; Zalatan *et al* also observed that two or three copies of *MS2* decreased the stability of gRNA (31). On the other hand, conjugating multiple aptamers to each RNA molecule causes the possibility of multiple NC-MCP proteins binding to one mRNA molecule, resulting in reduced packaging capacity. In addition to titrating the best aptamer copy numbers for efficient *SaCas9* mRNA packaging, we further introduced *HBB* 3′ UTR to enhance the mRNA stability and translatability. Differences in packaging plasmid modification and target mRNA configuration explained why we could obtain high particle yield, package more copies of mRNA per particle and obtain high genome editing efficiency.

One unanswered question is the mechanism for packaging *SaCas9* mRNA without aptamer (*SaCas9^0xMS2^*). It can only be packaged in the presence of aptamer-binding proteins (MCP or PCP). Two possibilities could explain the packaging of *MS2*-freee *SaCas9* mRNA in LVLPs: overexpressed cellular mRNA could be packaged into the LVLPs non-specifically (57), or *SaCas9* mRNA might have some RNA structures interacting with MCP or PCP. Although the mechanism for this aptamer-independent and aptamer-binding protein-dependent packaging of *SaCas9* mRNA is unknown at present, this is a useful observation since in cases where moderate Cas9 expression is preferred, we can package the aptamer-free *SaCas9* mRNA, instead of the one with *MS2* and *HBB* 3′UTR. The availability of a serial *SaCas9* mRNA LVLPs with different genome editing activities makes it possible to choose one most suited for a specific application. In addition to packaging mRNA encoding SaCas9 nuclease, this system should be able to package mRNAs coding for other editor proteins (e.g. spCas9, Cas12a), and for Cas9 without endonuclease activity (dCas9) for CRISPR interference (CRISPRi), CRISPR activation (CRISPRa), and base editing. Indeed, our preliminary data suggest that similar strategy can be used to package *SpCas9* and *Cas12a* (*cpf1*) mRNA (our unpublished results).

At the moment we could not rule out a minor contribution from possible residual plasmid DNA although we showed that it did not have a major role. Residual plasmid DNA has the possibility of integrating in genome and causing safety risks. Note that plasmid DNA might not be the only source of the background activities we observed (e.g. in Figure 2D); mRNA packaged non-specifically in LVLP or extracellular vesicles could also contribute to the observed activities. Nevertheless, the LVLP system is expected to have lower risk of DNA integration than AAV, IDLV and LV. Even if there is residual DNA in LVLP preparations, the issue of residual plasmid DNA is common to AAV, IDLV and LV vectors. It is observed that the genome delivered by IDLV integrated at a rate of 0.8% (58). Thus the genomes delivered by AAV, IDLV and LV have a much greater chance for integration than the RNAs delivered by our LVLPs.

Currently we only packaged *SaCas9* mRNA in LVLPs. Considering the recent observation of high prevalence of Cas9 immune responses within the adult human population (59,60), our transient delivery system will not only improve safety but also avoid possible immune response to the editor proteins, which could improve *in vivo* gene editing efficiency. Although transient expression of Cas9 is able to ensure transient function of the whole editing machinery, similarly packaging sgRNA or co-packaging *SaCas9* mRNA with sgRNA will make the system more convenient and efficient. We are currently exploring these possibilities in the lab.

In summary, the present work turns the widely used lentiviral vector into an efficient RNA delivery vehicle for transient expression. It will be useful for transient expression of not only editor proteins, but also other regulatory proteins (e.g. transcription factors) for transient gene regulation.

## DATA AVAILABILITY

All data that support the findings of this study are available in the paper and its Supplementary Information.

## Supplementary Material

Supplementary Data
