# Supplementary material for: Delivering SaCas9 mRNA by lentivirus-like bionanoparticles for transient expression and efficient genome editing
Source: Nucleic Acids Res. 2019 Feb 13;47(8):e44. doi: 10.1093/nar/gkz093 (PMC6486560; doi:10.1093/nar/gkz093)
Supplement: Supplementary Data [file nar_47_8_e44_s1.zip › supplementary data.docx]

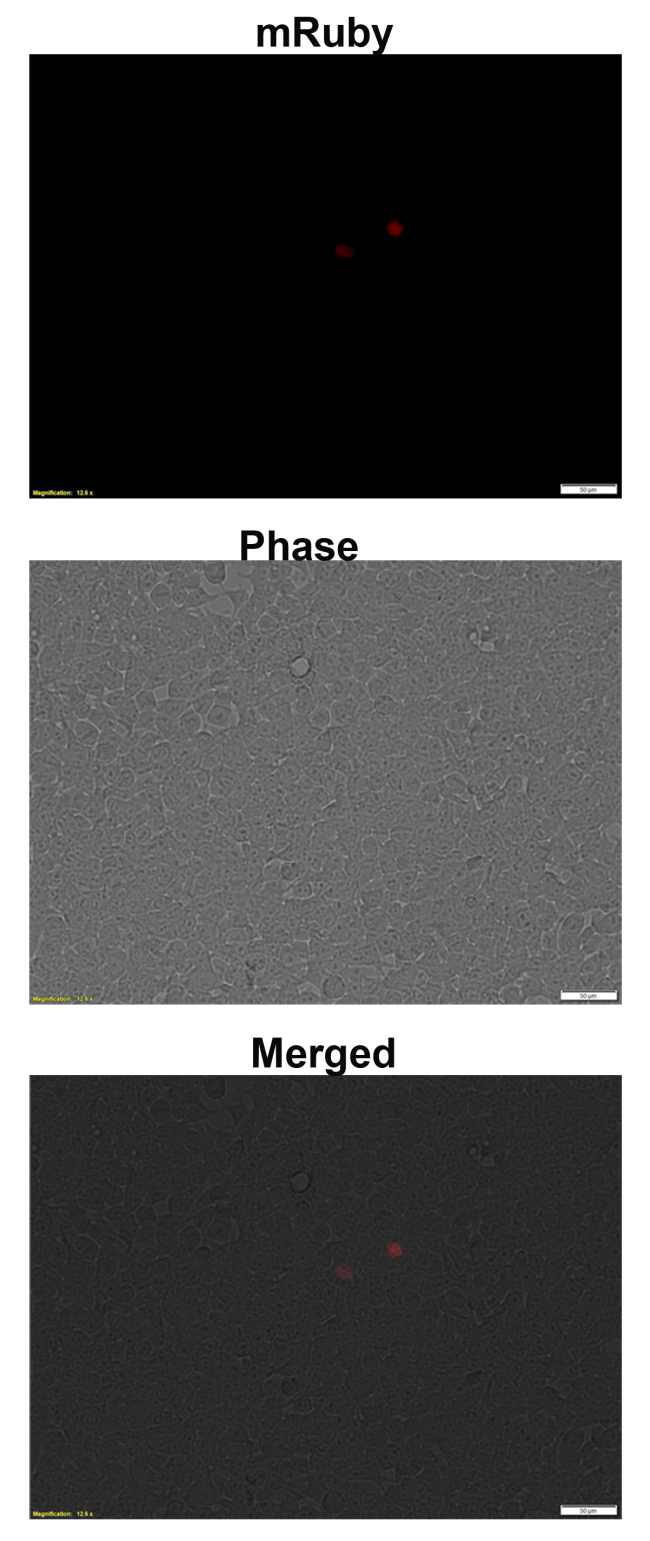


FigS1. Few mRuby-positive cells could be observed. The GFP-reporter cells were transduced with supernatant generated by replacing the packaging plasmid with the (nuclear located) mRuby expressing plasmid. Shown are images from one field of the cells treated with 534 µl of supernatant. Multiple fields were examined and 0-5 positive cells were observed per field. Three fields, with 2, 2 and 3 positive cells respectively, were estimated by imageJ to have 934 cells/field. The average positive rate was 0.0025%.





Fig.S2. Electron microscopy of NC-MCP and NC-PCP modified LVLPs. Shown below the images are the means ± SEM with sample numbers in parentheses. No statistical differences were observed between groups.


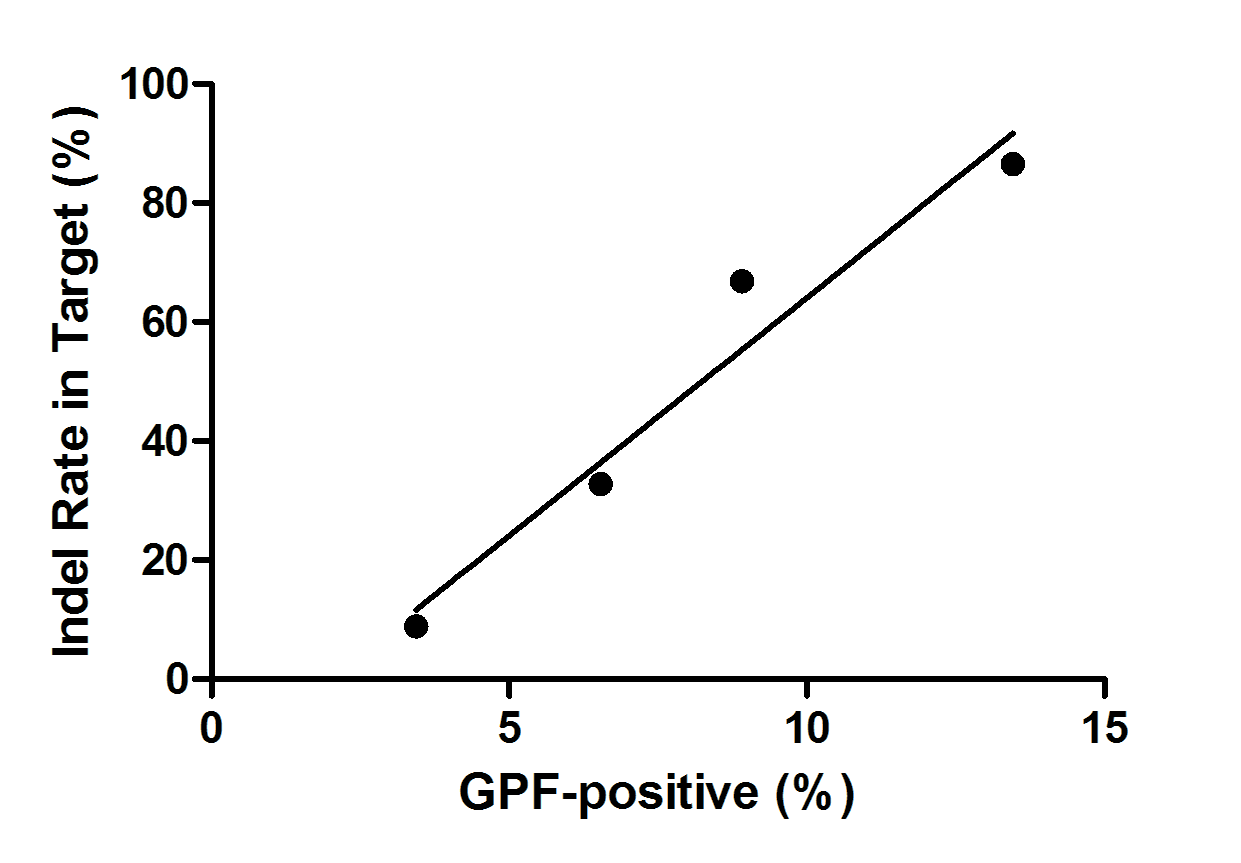


Fig.S3. Relationship between target sequence Indel rates and GFP-positive rates in GFP reporter cells. The four data points were from GFP-reporter cells transduced with *SaCas9*^1x^*^MS^*^2^ LVLPs (about 750 ng p24 for 1.25x10^6^ cells), SaCas9 AAV6 (10^4^ vg/cell), SaCas9 IDLV (about 750 ng p24 for 1.25x10^6^ cells), and *SaCas9*^1x^*^MS^*^2^-*HBB* 3’UTR (30 ng p24 for 2x5x10^4^ cells). The target sequences in the GFP-expression cassette were amplified and subjected to next generation sequencing. The Indel rates were plotted against the GFP-positive rates obtained by flow cytometry analysis.
