# Supplementary material for: Delivering SaCas9 mRNA by lentivirus-like bionanoparticles for transient expression and efficient genome editing
Source: Nucleic Acids Res. 2019 Feb 13;47(8):e44. doi: 10.1093/nar/gkz093 (PMC6486560; doi:10.1093/nar/gkz093)
Supplement: Supplementary Data [file nar_47_8_e44_s1.zip › Supplementary Table 2.docx]

**Supplementary Table 2. Sequence information for the study**

| SEQ ID NO | Sequence Type | Sequence |
| --- | --- | --- |
| SEQ ID NO:1 | HIV-1 Nucleocapsid (NC) DNA Sequence | ATACAGAAAGGCAATTTTAGGAACCAAAGAAAGACTGTTAAGTGTTTCAATTGTGGCAAAGAAGGGCACATAGCCAAAAATTGCAGGGCCCCTAGGAAAAAGGGCTGTTGGAAATGTGGAAAGGAAGGACACCAAATGAAAGATTGTACTGAGAGACAGGCTAAT |
| SEQ ID NO:2 | HIV-1 Nucleocapsid (NC) Amino Acid Sequence | IQKGNFRNQRKTVKCFNCGKEGHIAKNCRAPRKKGCWKCGKEGHQMKDCTERQAN |
| SEQ ID NO:3 | HIV-1 Matrix protein (MA) DNA Sequence | atgggtgcgagagcgtcagtattaagcgggggagaattagatcgatgggaaaaaattcggttaaggccagggggaaagaaaaaatataaattaaaacatatagtatgggcaagcagggagctagaacgattcgcagttaatcctggcctgttagaaacatcagaaggctgtagacaaatactgggacagctacaaccatcccttcagacaggatcagaagaacttagatcattatataatacagtagcaaccctctattgtgtgcatcaaaggatagagataaaagacaccaaggaagctttagacaagatagaggaagagcaaaacaaaagtaagaaaaaagcacagcaagcagcagctgacacaggacacagcaatcaggtcagccaaaattac |
| SEQ ID NO:4 | HIV-1 Matrix protein (MA) Amino Acid Sequence | GARASVLSGGELDRWEKIRLRPGGKKKYKLKHIVWASRELERFAVNPGLLETSEGCRQILGQLQPSLQTGSEELRSLYNTVATLYCVHQRIEIKDTKEALDKIEEEQNKSKKKAQQAAADTGHSNQVSQNY |
| SEQ ID NO:5 | HIV-1 Viral Protein (VPR) DNA Sequence | ATGGAACAAGCCCCAGAAGACCAGGGACCGCAGAGGGAACCATACAATGAATGGACACTAGAACTTTTAGAGGAACTCAAGCGGGAAGCAGTCAGACACTTTCCTAGACCATGGCTTCATGGCTTAGGACAACATATCTATGAAACCTATGGAGATACTTGGACGGGGGTGGAAGCTATAATAAGAATTCTGCAACGACTACTGTTTGTCCATTTCAGAATTGGGTGCCAGCATAGCCGAATAGGCATTCTAAGACAGAGAAGAGCAAGAAATGGAGCCAGTAGATCCTAA |
| SEQ ID NO:6 | HIV-1 Viral Protein (VPR) Amino Acid Sequence | MEQAPEDQGPQREPYNEWTLELLEELKREAVRHFPRPWLHGLGQHIYETYGDTWTGVEAIIRILQRLLFVHFRIGCQHSRIGILRQRRARNGASRS |
| SEQ ID NO:7 | HIV-1 Negative Regulatory Factor (NEF) DNA Sequence with codon changes to enhance packaging in the virus core (G3C, V153L, and E177G mutations; underlined) | atgggtTgcaagtggtcaaaaagtagtgtgattggatggcctgctgtaagggaaagaatgagacgagctgagccagcagcagatggggtgggagcagtatctcgagacctagaaaaacatggagcaatcacaagtagcaatacagcagctaacaatgctgcttgtgcctggctagaagcacaagaggaggaagaggtgggttttccagtcacacctcaggtacctttaagaccaatgacttacaaggcagctgtagatcttagccactttttaaaagaaaaggggggactggaagggctaattcactcccaaagaagacaagatatccttgatctgtggatctaccacacacaaggctacttccctgattggcagaactacacaccagggccaggggtcagatatccactgacctttggatggtgctacaagctagtaccagttgagccagataagCtGgaagaggccaataaaggagagaacaccagcttgttacaccctgtgagcctgcatggaatggatgaccctgGAagagaagtgttagagtggaggtttgacagccgcctagcatttcatcacgtggcccgagagctgcatccggagtacttcaagaactgc (The letters in capitals are changed to code for the changes explained in seq ID. 8. |
| SEQ ID NO:8 | HIV-1 Negative Regulatory Factor (NEF) Amino Acid Sequence with mutation to enhance packaging in the virus core (G3C, V153L, and E177G mutations; underlined) | MGCKWSKSSVIGWPAVRERMRRAEPAADGVGAVSRDLEKHGAITSSNTAANNAACAWLEAQEEEEVGFPVTPQVPLRPMTYKAAVDLSHFLKEKGGLEGLIHSQRRQDILDLWIYHTQGYFPDWQNYTPGPGVRYPLTFGWCYKLVPVEPDKLEEANKGENTSLLHPVSLHGMDDPGREVLEWRFDSRLAFHHVARELHPEYFKNC |
| SEQ ID NO:9 | MS2 coat protein (MCP) DNA Sequence | ATGGCTTCTAACTTTACTCAGTTCGTTCTCGTCGACAATGGCGGAACTGGCGACGTGACTGTCGCCCCAAGCAACTTCGCTAACGGGATCGCTGAATGGATCAGCTCTAACTCGCGTTCACAGGCTTACAAAGTAACCTGTAGCGTTCGTCAGAGCTCTGCGCAGAATCGCAAATACACCATCAAAGTCGAGGTGCCTAAAGGCGCCTGGCGTTCGTACTTAAATATGGAACTAACCATTCCAATTTTCGCCACGAATTCCGACTGCGAGCTTATTGTTAAGGCAATGCAAGGTCTCCTAAAAGATGGAAACCCGATTCCCTCAGCAATCGCAGCAAACTCCGGCATCTAC |
| SEQ ID NO:10 | MS2 coat protein (MCP) Amino Acid Sequence | MASNFTQFVLVDNGGTGDVTVAPSNFANGIAEWISSNSRSQAYKVTCSVRQSSAQNRKYTIKVEVPKGAWRSYLNMELTIPIFATNSDCELIVKAMQGLLKDGNPIPSAIAANSGIY |
| SEQ ID NO:11 | PP7 coat protein (PCP) DNA Sequence | tccaaaacaatagtcctctccgtaggggaggcaacacggactttgaccgaaatccagtcaaccgctgaccgacaaatctttgaagagaaagtagggcctcttgtgggccgactgcgcttgactgcaagcttgcgacaaaacggcgcaaagactgcctatagggtcaaccttaaactcgaccaagccgacgtggtcgatagcggtctccctaaggttcggtatacgcaggtctggagtcatgacgtaacaatcgtagcaaacagcacagaagcctcccgaaaaagcctctacgatctgacgaaatccttggtggctacgtcacaggtggaagacctcgttgtcaaccttgtacctctgggtcga |
| SEQ ID NO:12 | PP7 coat protein (PCP) Amino Acid Sequence | SKTIVLSVGEATRTLTEIQSTADRQIFEEKVGPLVGRLRLTASLRQNGAKTAYRVNLKLDQADVVDSGLPKVRYTQVWSHDVTIVANSTEASRKSLYDLTKSLVATSQVEDLVVNLVPLGR |
| SEQ ID NO:17 | MS2 aptamer sequence (RNA) | ACAUGAGGAUCACCCAUGU |
| SEQ ID NO:18 | MS2 aptamer sequence (DNA) | ACATGAGGATCACCCATGT |
| SEQ ID NO:19 | PP7 aptamer sequence (RNA) | GGAGCAGACGAUAUGGCGUCGCUCC |
| SEQ ID NO:20 | PP7 aptamer sequence (DNA) | GGAGCAGACGATATGGCGTCGCTCC |
| SEQ ID NO:25 | human beta hemoglobin (HBB) 3’ UTR (DNA) | gctcgctttcttgctgtccaatttctattaaaggttcctttgttccctaagtccaactactaaactgggggatattatgaagggccttgagcatctggattctgcctaataaaaaacatttattttcattgc |
| SEQ ID NO:26 | human beta hemoglobin (HBB) 3’ UTR (RNA) | gcucgcuuucuugcuguccaauuucuauuaaagguuccuuuguucccuaaguccaacuacuaaacugggggauauuaugaagggccuugagcaucuggauucugccuaauaaaaaacauuuauuuucauugc |
| SEQ ID NO:27 | human hemoglobin alpha (HBA) 5’ UTR (DNA) | Ctcttctggtccccacagactcagagagaa c |
| SEQ ID NO:28 | SaCas9-2576F | AAACCGGGAACTACCTGACC |
| SEQ ID NO:29 | SaCas9- 2713R | TCACGACCTTGTTTCTGCTG |
| SEQ ID NO:30 | SgRNA-F1 | GAGTAACGGCAGACTTCTCCA |
| SEQ ID NO:31 | sgRNA-R1 | CGGCATTTTGCCTTGTTTAAG |
| SEQ ID NO:32 | EGFP-RT-F | CAGTGCTTCAGCCGCTACCC |
| SEQ ID NO:33 | EGFP-RT-R | AGCTCGATGCGGTTCACCAG |
| SEQ ID NO:34 | HBB-mut-F1 | TCGTCGGCAGCGTCAGATGTGTATAAGAGACAGTGTTCACTAGCAACCTCAAACAG |
| SEQ ID NO:35 | HBB-mut-F2 | TCGTCGGCAGCGTCAGATGTGTATAAGAGACAGATGTTCACTAGCAACCTCAAACAG |
| SEQ ID NO:36 | HBB-mut-F3 | TCGTCGGCAGCGTCAGATGTGTATAAGAGACAGGATGTTCACTAGCAACCTCAAACAG |
| SEQ ID NO:37 | HBB-mut-F4 | TCGTCGGCAGCGTCAGATGTGTATAAGAGACAGCGATGTTCACTAGCAACCTCAAACAG |
| SEQ ID NO:38 | HBB-mut-F5 | TCGTCGGCAGCGTCAGATGTGTATAAGAGACAGTCGATGTTCACTAGCAACCTCAAACAG |
| SEQ ID NO:39 | HBB-mut-F6 | TCGTCGGCAGCGTCAGATGTGTATAAGAGACAGATCGATGTTCACTAGCAACCTCAAACAG |
| SEQ ID NO:40 | HBB-mut-R1 | GTCTCGTGGGCTCGGAGATGTGTATAAGAGACAGTCCAATAGGCAGAGAGAGTCAGTG |
| SEQ ID NO:41 | Oligo 1xloop-F | TTACGCTTAAGAATTCTAGAAAACATGAGGATCACCCATGTCTGCAGGTCGACTCTAGAAAATTCCTAGAGCTCG |
| SEQ ID NO:42 | Oligo 1xloop-R | CGAGCTCTAGGAATTTTCTAGAGTCGACCTGCAGACATGGGTGATCCTCATGTTTTCTAGAATTCTTAAGCGTAA |
| SEQ ID NO:43 | Oligo 2xloop-F | TTACGCTTAAGAATTCTAGAAAACATGAGGATCACCCATGTCTGCAGGTCGACTCTAGAAAACATGAGGATCACCCATGTCTGCAAAATTCCTAGAGCTCG |
| SEQ ID NO:44 | Oligo 2xloop-R | CGAGCTCTAGGAATTTTGCAGACATGGGTGATCCTCATGTTTTCTAGAGTCGACCTGCAGACATGGGTGATCCTCATGTTTTCTAGAATTCTTAAGCGTAA |
| SEQ ID NO:45 | Oligo 3xloop-F | TTACGCTTAAGAATTCTAGAAAACATGAGGATCACCCATGTCTGCAGGTCGACTCTAGAAAACATGAGGATCACCCATGTCTGCAAAATTCTAGAAAACAT |
| SEQ ID NO:46 | Oligo 3xloop-R | ATGTTTTCTAGAATTTTGCAGACATGGGTGATCCTCATGTTTTCTAGAGTCGACCTGCAGACATGGGTGATCCTCATGTTTTCTAGAATTCTTAAGCGTAA |
| SEQ ID NO:47 | Oligo Sickle-g2F | CACCGCCCTGTGGGGCAAGGTGAAC |
| SEQ ID NO:48 | Oligo Sickle-g2R | AAACGTTCACCTTGCCCCACAGGGC |
| SEQ ID NO:49 | Oligo Sickle-g1F | CACCGAGTAACGGCAGACTTCTCCAC |
| SEQ ID NO:50 | Sickle-g1R | AAACGTGGAGAAGTCTGCCGTTACTC |
| SEQ ID NO:51 | Oligo PP7-F | TTACGCTTAAGAATTCGGAGCAGACGATATGGCGTCGCTCCGAATTCCTAGAGCTCG |
| SEQ ID NO:52 | Oligo PP7-R | CGAGCTCTAGGAATTCGGAGCGACGCCATATCGTCTGCTCCGAATTCTTAAGCGTAA |
| SEQ ID NO:53 | Oligo Sickle-g1-LV-F | caccGAGTAACGGCAGACTTCTCCAC |
| SEQ ID NO:54 | Oligo sickle-g1-LV-R | gaacGTGGAGAAGTCTGCCGTTACTC |
| SEQ ID NO:55 | Primer Sickle-g1HD-F | TTGCAATGATCTCGAGGGCCTATTTCCCATGATTC |
| SEQ ID NO:56 | Primer Sickle-g1HD-R | CTGCGGCCGCTCTAGAAAAATCTCGCCAACAAGTTG |
| SEQ ID NO:57 | oligo HBB-tem-F | CATGGTGCATCTGACACCTGTGGAGAAGTCTGCCGTTACTGCCCTGTGGGGCAAGGTGAACGTGGATGAAGTTGGTGGTGAGGCC |
| SEQ ID NO:58 | oligo HBB-tem-R | CAGGGCCTCACCACCAACTTCATCCACGTTCACCTTGCCCCACAGGGCAGTAACGGCAGACTTCTCCACAGGTGTCAGATGCAC |
| SEQ ID NO:59 | HBB-LT-F | CTTCTGATTTTCTAGATTGTGTAATCGTAGTTTCAGAG |
| SEQ ID NO:60 | HBB-LT-R | GCTTGATATCGAATTAAAAAATCTCGCCAACAAGTTGAC |
| SEQ ID NO:61 | pCDNA3.1/MS2x2-VPR DNA Oligo Insert | *GCTAGCcaccATGGCTTCTAACTTTACTCAGTTCGTTCTCGTCGACAATGGCGGAACTGGCGACGTGACTGTCGCCCCAAGCAACTTCGCTAACGGGATCGCTGAATGGATCAGCTCTAACTCGCGTTCACAGGCTTACAAAGTAACCTGTAGCGTTCGTCAGAGCTCTGCGCAGAATCGCAAATACACCATCAAAGTCGAGGTGCCTAAAGGCGCCTGGCGTTCGTACTTAAATATGGAACTAACCATTCCAATTTTCGCCACGAATTCCGACTGCGAGCTTATTGTTAAGGCAATGCAAGGTCTCCTAAAAGATGGAAACCCGATTCCCTCAGCAATCGCAGCAAACTCCGGCATCTACGGTGGTGGAGGAGGAATGGCGTCCAATTTCACGCAGTTCGTCCTGGTTGACAACGGGGGGACTGGGGACGTTACGGTCGCTCCGAGCAACTTTGCCAATGGTATTGCGGAGTGGATTTCTTCTAATTCACGGTCCCAAGCTTACAAAGTGACCTGTTCCGTGCGGCAAAGTTCTGCTCAGAATAGAAAGTACACTATAAAGGTCGAAGTCCCTAAGGGGGCCTGGCGATCATATCTCAATATGGAGCTTACCATCCCAATATTTGCCACTAATTCTGATTGTGAATTGATTGTCAAAGCAATGCAAGGACTCTTGAAAGACGGAAACCCAATCCCCAGCGCAATCGCAGCCAACTCCGGTATATACGGAGGTGGTGGAGGAATGGAACAAGCCCCAGAAGACCAGGGACCGCAGAGGGAACCATACAATGAATGGACACTAGAACTTTTAGAGGAACTCAAGCGGGAAGCAGTCAGACACTTTCCTAGACCATGGCTTCATGGCTTAGGACAACATATCTATGAAACCTATGGAGATACTTGGACGGGGGTGGAAGCTATAATAAGAATTCTGCAACGACTACTGTTTGTCCATTTCAGAATTGGGTGCCAGCATAGCCGAATAGGCATTCTAAGACAGAGAAGAGCAAGAAATGGAGCCAGTAGATCCTAAgcggccgc* |
| SEQ ID NO:62 | pCDNA3.1/MS2x2-VPR Oligo Insert Amino Acid Sequence | *MASNFTQFVLVDNGGTGDVTVAPSNFANGIAEWISSNSRSQAYKVTCSVRQSSAQNRKYTIKVEVPKGAWRSYLNMELTIPIFATNSDCELIVKAMQGLLKDGNPIPSAIAANSGIYGGGGGMASNFTQFVLVDNGGTGDVTVAPSNFANGIAEWISSNSRSQAYKVTCSVRQSSAQNRKYTIKVEVPKGAWRSYLNMELTIPIFATNSDCELIVKAMQGLLKDGNPIPSAIAANSGIYGGGGGMEQAPEDQGPQREPYNEWTLELLEELKREAVRHFPRPWLHGLGQHIYETYGDTWTGVEAIIRILQRLLFVHFRIGCQHSRIGILRQRRARNGASRS* |
| SEQ ID NO:63 | pCDNA3.1/NEF-MS2x2 DNA Oligo Insert | *GCTAGCcaccatgggtTgcaagtggtcaaaaagtagtgtgattggatggcctgctgtaagggaaagaatgagacgagctgagccagcagcagatggggtgggagcagtatctcgagacctagaaaaacatggagcaatcacaagtagcaatacagcagctaacaatgctgcttgtgcctggctagaagcacaagaggaggaagaggtgggttttccagtcacacctcaggtacctttaagaccaatgacttacaaggcagctgtagatcttagccactttttaaaagaaaaggggggactggaagggctaattcactcccaaagaagacaagatatccttgatctgtggatctaccacacacaaggctacttccctgattggcagaactacacaccagggccaggggtcagatatccactgacctttggatggtgctacaagctagtaccagttgagccagataagCtGgaagaggccaataaaggagagaacaccagcttgttacaccctgtgagcctgcatggaatggatgaccctgGAagagaagtgttagagtggaggtttgacagccgcctagcatttcatcacgtggcccgagagctgcatccggagtacttcaagaactgcGGAGGTGGTGGAGGAATGGCTTCTAACTTTACTCAGTTCGTTCTCGTCGACAATGGCGGAACTGGCGACGTGACTGTCGCCCCAAGCAACTTCGCTAACGGGATCGCTGAATGGATCAGCTCTAACTCGCGTTCACAGGCTTACAAAGTAACCTGTAGCGTTCGTCAGAGCTCTGCGCAGAATCGCAAATACACCATCAAAGTCGAGGTGCCTAAAGGCGCCTGGCGTTCGTACTTAAATATGGAACTAACCATTCCAATTTTCGCCACGAATTCCGACTGCGAGCTTATTGTTAAGGCAATGCAAGGTCTCCTAAAAGATGGAAACCCGATTCCCTCAGCAATCGCAGCAAACTCCGGCATCTACGGTGGTGGAGGAGGAATGGCGTCCAATTTCACGCAGTTCGTCCTGGTTGACAACGGGGGGACTGGGGACGTTACGGTCGCTCCGAGCAACTTTGCCAATGGTATTGCGGAGTGGATTTCTTCTAATTCACGGTCCCAAGCTTACAAAGTGACCTGTTCCGTGCGGCAAAGTTCTGCTCAGAATAGAAAGTACACTATAAAGGTCGAAGTCCCTAAGGGGGCCTGGCGATCATATCTCAATATGGAGCTTACCATCCCAATATTTGCCACTAATTCTGATTGTGAATTGATTGTCAAAGCAATGCAAGGACTCTTGAAAGACGGAAACCCAATCCCCAGCGCAATCGCAGCCAACTCCGGTATATACTGAgcggccgc* |
| SEQ ID NO:64 | pCDNA3.1/NEF-MS2x2 Oligo Insert Amino Acid Sequence | TMGCKWSKSSVIGWPAVRERMRRAEPAADGVGAVSRDLEKHGAITSSNTAANNAACAWLEAQEEEEVGFPVTPQVPLRPMTYKAAVDLSHFLKEKGGLEGLIHSQRRQDILDLWIYHTQGYFPDWQNYTPGPGVRYPLTFGWCYKLVPVEPDKLEEANKGENTSLLHPVSLHGMDDPGREVLEWRFDSRLAFHHVARELHPEYFKNCGGGGGMASNFTQFVLVDNGGTGDVTVAPSNFANGIAEWISSNSRSQAYKVTCSVRQSSAQNRKYTIKVEVPKGAWRSYLNMELTIPIFATNSDCELIVKAMQGLLKDGNPIPSAIAANSGIYGGGGGMASNFTQFVLVDNGGTGDVTVAPSNFANGIAEWISSNSRSQAYKVTCSVRQSSAQNRKYTIKVEVPKGAWRSYLNMELTIPIFATNSDCELIVKAMQGLLKDGNPIPSAIAANSGIY |
| SEQ ID NO:65 | pMDLg/pRRE-D64V-NC-MS2x1 DNA Oligo Insert | cctaggaaaaagggctgttggaaatgtggaaaggaaggacaccaaatgaaagattgttccggtggaggtggatccggtggaggttccatggcgtccaatttcacgcagttcgtcctggttgacaacggggggactggggacgttacggtcgctccgagcaactttgccaatggtattgcggagtggatttcttctaattcacggtcccaagcttacaaagtgacctgttccgtgcggcaaagttctgctcagaatagaaagtacactataaaggtcgaagtccctaagggggcctggcgatcatatctcaatatggagcttaccatcccaatatttgccactaattctgattgtgaattgattgtcaaagcaatgcaaggactcttgaaagacggaaacccaatccccagcgcaatcgcagccaactccggtatatactccggaggtggaggtggaactgagagacaggctaattttttagggaagatctggccttcccacaagggaaggccagggaattttcttcagagcagaccagagccaacagccccaccagaagagagcttcaggtttggggaagagacaacaactccctctcagaagcaggagccgatagacaaggaactgtatcctttagcttccctcagatcactctttggcagcgacccctcgtcacaataaagataggggggcaattaaaggaagctctattagatacaggagcagatgatacagtattagaagaaatgaatttgccaggaagatggaaaccaaaaatgatagggggaattggaggttttatcaaagtaagacagtatgatcagatactcatagaaatctgcggacataaagctataggtacagtattagtaggacctacacctgtcaacataattggaagaaatctgttgactcagattggctgcactttaaattttcccattagtcctattgagactgtaccagtaaaattaaagccaggaatggatggcccaaaagttaaacaatggccattgacagaagaaaaaataaaagcattagtagaaatttgtacagaaatggaaaaggaaggaaaaatttcaaaaattgggcctgaaaatccatacaatactccagtatttgccataaagaaaaaagacagtactaaatggagaaaattagtagatttcagagaacttaataagagaactcaagatttctgggaagttcaattaggaataccacatcctgcagg |
| SEQ ID NO:66 | pMDLg/pRRE-D64V-NC-MS2x1 Oligo Insert Amino Acid Sequence | PRKKGCWKCGKEGHQMKDCSGGGGSGGGSMASNFTQFVLVDNGGTGDVTVAPSNFANGIAEWISSNSRSQAYKVTCSVRQSSAQNRKYTIKVEVPKGAWRSYLNMELTIPIFATNSDCELIVKAMQGLLKDGNPIPSAIAANSGIYSGGGGGTERQANFLGKIWPSHKGRPGNFLQSRPEPTAPPEESFRFGEETTTPSQKQEPIDKELYPLASLRSLFGSDPSSQ |
| SEQ ID NO:67 | pMDLg/pRRE-D64V-NC-MS2x2 DNA Oligo Insert | CCTAGGAAAAAGGGCTGTTGGAAATGTGGAAAGGAAGGACACCAAATGAAAGATTGTTCCGGTGGAGGTGGATCCATGGCTTCTAACTTTACTCAGTTCGTTCTCGTCGACAATGGCGGAACTGGCGACGTGACTGTCGCCCCAAGCAACTTCGCTAACGGGATCGCTGAATGGATCAGCTCTAACTCGCGTTCACAGGCTTACAAAGTAACCTGTAGCGTTCGTCAGAGCTCTGCGCAGAATCGCAAATACACCATCAAAGTCGAGGTGCCTAAAGGCGCCTGGCGTTCGTACTTAAATATGGAACTAACCATTCCAATTTTCGCCACGAATTCCGACTGCGAGCTTATTGTTAAGGCAATGCAAGGTCTCCTAAAAGATGGAAACCCGATTCCCTCAGCAATCGCAGCAAACTCCGGCATCTACGGATCCGGTGGAGGTTCCATGGCGTCCAATTTCACGCAGTTCGTCCTGGTTGACAACGGGGGGACTGGGGACGTTACGGTCGCTCCGAGCAACTTTGCCAATGGTATTGCGGAGTGGATTTCTTCTAATTCACGGTCCCAAGCTTACAAAGTGACCTGTTCCGTGCGGCAAAGTTCTGCTCAGAATAGAAAGTACACTATAAAGGTCGAAGTCCCTAAGGGGGCCTGGCGATCATATCTCAATATGGAGCTTACCATCCCAATATTTGCCACTAATTCTGATTGTGAATTGATTGTCAAAGCAATGCAAGGACTCTTGAAAGACGGAAACCCAATCCCCAGCGCAATCGCAGCCAACTCCGGTATATACTCCGGAGGTGGAGGTGGAACTGAGAGACAGGCTAATTTTTTAGGGAAGATCTGGCCTTCCCACAAGGGAAGGCCAGGGAATTTTCTTCAGAGCAGACCAGAGCCAACAGCCCCACCAGAAGAGAGCTTCAGGTTTGGGGAAGAGACAACAACTCCCTCTCAGAAGCAGGAGCCGATAGACAAGGAACTGTATCCTTTAGCTTCCCTCAGATCACTCTTTGGCAGCGACCCCTCGTCACAATAAAGATAGGGGGGCAATTAAAGGAAGCTCTATTAGATACAGGAGCAGATGATACAGTATTAGAAGAAATGAATTTGCCAGGAAGATGGAAACCAAAAATGATAGGGGGAATTGGAGGTTTTATCAAAGTAAGACAGTATGATCAGATACTCATAGAAATCTGCGGACATAAAGCTATAGGTACAGTATTAGTAGGACCTACACCTGTCAACATAATTGGAAGAAATCTGTTGACTCAGATTGGCTGCACTTTAAATTTTCCCATTAGTCCTATTGAGACTGTACCAGTAAAATTAAAGCCAGGAATGGATGGCCCAAAAGTTAAACAATGGCCATTGACAGAAGAAAAAATAAAAGCATTAGTAGAAATTTGTACAGAAATGGAAAAGGAAGGAAAAATTTCAAAAATTGGGCCTGAAAATCCATACAATACTCCAGTATTTGCCATAAAGAAAAAAGACAGTACTAAATGGAGAAAATTAGTAGATTTCAGAGAACTTAATAAGAGAACTCAAGATTTCTGGGAAGTTCAATTAGGAATACCACATCCTGCAGG |
| SEQ ID NO:68 | pMDLg/pRRE-D64V-NC-MS2x2 Oligo Insert Amino Acid Sequence | PRKKGCWKCGKEGHQMKDCSGGGGSMASNFTQFVLVDNGGTGDVTVAPSNFANGIAEWISSNSRSQAYKVTCSVRQSSAQNRKYTIKVEVPKGAWRSYLNMELTIPIFATNSDCELIVKAMQGLLKDGNPIPSAIAANSGIYGSGGGSMASNFTQFVLVDNGGTGDVTVAPSNFANGIAEWISSNSRSQAYKVTCSVRQSSAQNRKYTIKVEVPKGAWRSYLNMELTIPIFATNSDCELIVKAMQGLLKDGNPIPSAIAANSGIYSGGGGGTERQANFLGKIWPSHKGRPGNFLQSRPEPTAPPEESFRFGEETTTPSQKQEPIDKELYPLASLRSLFGSDPSSQ |
| SEQ ID NO:69 | pMDLg/pRRE-D64V-NC-PP7x1 DNA Oligo Insert | cctaggaaaaagggctgttggaaatgtggaaaggaaggacaccaaatgaaagattgttccggtggaggtggatcctccaaaacaatagtcctctccgtaggggaggcaacacggactttgaccgaaatccagtcaaccgctgaccgacaaatctttgaagagaaagtagggcctcttgtgggccgactgcgcttgactgcaagcttgcgacaaaacggcgcaaagactgcctatagggtcaaccttaaactcgaccaagccgacgtggtcgatagcggtctccctaaggttcggtatacgcaggtctggagtcatgacgtaacaatcgtagcaaacagcacagaagcctcccgaaaaagcctctacgatctgacgaaatccttggtggctacgtcacaggtggaagacctcgttgtcaaccttgtacctctgggtcggtccggaggtggaggtggaactgagagacaggctaattttttagggaagatctggccttcccacaagggaaggccagggaattttcttcagagcagaccagagccaacagccccaccagaagagagcttcaggtttggggaagagacaacaactccctctcagaagcaggagccgatagacaaggaactgtatcctttagcttccctcagatcactctttggcagcgacccctcgtcacaataaagataggggggcaattaaaggaagctctattagatacaggagcagatgatacagtattagaagaaatgaatttgccaggaagatggaaaccaaaaatgatagggggaattggaggttttatcaaagtaagacagtatgatcagatactcatagaaatctgcggacataaagctataggtacagtattagtaggacctacacctgtcaacataattggaagaaatctgttgactcagattggctgcactttaaattttcccattagtcctattgagactgtaccagtaaaattaaagccaggaatggatggcccaaaagttaaacaatggccattgacagaagaaaaaataaaagcattagtagaaatttgtacagaaatggaaaaggaaggaaaaatttcaaaaattgggcctgaaaatccatacaatactccagtatttgccataaagaaaaaagacagtactaaatggagaaaattagtagatttcagagaacttaataagagaactcaagatttctgggaagttcaattaggaataccacatcctgcagg |
| SEQ ID NO:70 | pMDLg/pRRE-D64V-NC-PP7x1 Oligo Insert Amino Acid Sequence | PRKKGCWKCGKEGHQMKDCSGGGGSSKTIVLSVGEATRTLTEIQSTADRQIFEEKVGPLVGRLRLTASLRQNGAKTAYRVNLKLDQADVVDSGLPKVRYTQVWSHDVTIVANSTEASRKSLYDLTKSLVATSQVEDLVVNLVPLGRSGGGGGTERQANFLGKIWPSHKGRPGNFLQSRPEPTAPPEESFRFGEETTTPSQKQEPIDKELYPLASLRSLFGSDPSSQ |
| SEQ ID NO:71 | pMDLg/pRRE-D64V-MA-MS2x2 DNA Oligo Insert | CACGTGAGATCTGAATTCGAGATCTGCCGCCGCCATGGGTGCGAGAGCGTCAGTATTAAGCGGGGGAGAATTAGATCGATGGGAAAAAATTCGGTTAAGGCCAGGGGGAAAGAAAAAATATAAATTAAAACATATAGTATGGGCAAGCAGGGAGCTAGAACGAGGAGGTGGTGGAGGAATGGCTTCTAACTTTACTCAGTTCGTTCTCGTCGACAATGGCGGAACTGGCGACGTGACTGTCGCCCCAAGCAACTTCGCTAACGGGATCGCTGAATGGATCAGCTCTAACTCGCGTTCACAGGCTTACAAAGTAACCTGTAGCGTTCGTCAGAGCTCTGCGCAGAATCGCAAATACACCATCAAAGTCGAGGTGCCTAAAGGCGCCTGGCGTTCGTACTTAAATATGGAACTAACCATTCCAATTTTCGCCACGAATTCCGACTGCGAGCTTATTGTTAAGGCAATGCAAGGTCTCCTAAAAGATGGAAACCCGATTCCCTCAGCAATCGCAGCAAACTCCGGCATCTACGGTGGTGGAGGAGGAATGGCGTCCAATTTCACGCAGTTCGTCCTGGTTGACAACGGGGGGACTGGGGACGTTACGGTCGCTCCGAGCAACTTTGCCAATGGTATTGCGGAGTGGATTTCTTCTAATTCACGGTCCCAAGCTTACAAAGTGACCTGTTCCGTGCGGCAAAGTTCTGCTCAGAATAGAAAGTACACTATAAAGGTCGAAGTCCCTAAGGGGGCCTGGCGATCATATCTCAATATGGAGCTTACCATCCCAATATTTGCCACTAATTCTGATTGTGAATTGATTGTCAAAGCAATGCAAGGACTCTTGAAAGACGGAAACCCAATCCCCAGCGCAATCGCAGCCAACTCCGGTATATACCAGGTCAGCCAAAATTACCCTATAGTGCAGAACATCCAGGGGCAAATGGTACATCAGGCCATATCACCTAGAACTTTAAATGCATGGGTAAAAGTAGTAGAAGAGAAGGCTTTCAGCCCAGAAGTGATACCCATGTTTTCAGCATTATCAGAAGGAGCCACCCCACAAGATTTAAACACCATGCTAAACACAGTGGGGGGACATCAAGCAGCCATGCAAATGTTAAAAGAGACCATCAATGAGGAAGCTGCAGAATGGGATAGAGTGCATCCAGTGCATGC |
| SEQ ID NO:72 | pMDLg/pRRE-D64V-MA-MS2x2 Oligo Insert Amino Acid Sequence | MGARASVLSGGELDRWEKIRLRPGGKKKYKLKHIVWASRELERGGGGGMASNFTQFVLVDNGGTGDVTVAPSNFANGIAEWISSNSRSQAYKVTCSVRQSSAQNRKYTIKVEVPKGAWRSYLNMELTIPIFATNSDCELIVKAMQGLLKDGNPIPSAIAANSGIYGGGGGMASNFTQFVLVDNGGTGDVTVAPSNFANGIAEWISSNSRSQAYKVTCSVRQSSAQNRKYTIKVEVPKGAWRSYLNMELTIPIFATNSDCELIVKAMQGLLKDGNPIPSAIAANSGIYQVSQNYPIVQNIQGQMVHQAISPRTLNAWVKVVEEKAFSPEVIPMFSALSEGATPQDLNTMLNTVGGHQAAMQMLKETINEEAAEWDRVHPVH |
| SEQ ID NO:73 | pMDLg/pRRE-D64V-MA-PP7x1 DNA Oligo Insert | Cacgtgagatctgaattcgagatctgccgccgccatgggtgcgagagcgtcagtattaagcgggggagaattagatcgatgggaaaaaattcggttaaggccagggggaaagaaaaaatataaattaaaacatatagtatgggcaagcagggagctagaacgaggaggtggtggaggaatggcttctaactttactcagttcgttctcgtcgactccaaaacaatagtcctctccgtaggggaggcaacacggactttgaccgaaatccagtcaaccgctgaccgacaaatctttgaagagaaagtagggcctcttgtgggccgactgcgcttgactgcaagcttgcgacaaaacggcgcaaagactgcctatagggtcaaccttaaactcgaccaagccgacgtggtcgatagcggtctccctaaggttcggtatacgcaggtctggagtcatgacgtaacaatcgtagcaaacagcacagaagcctcccgaaaaagcctctacgatctgacgaaatccttggtggctacgtcacaggtggaagacctcgttgtcaaccttgtacctctgggtcgaaaccaggtcagccaaaattaccctatagtgcagaacatccaggggcaaatggtacatcaggccatatcacctagaactttaaatgcatgggtaaaagtagtagaagagaaggctttcagcccagaagtgatacccatgttttcagcattatcagaaggagccaccccacaagatttaaacaccatgctaaacacagtggggggacatcaagcagccatgcaaatgttaaaagagaccatcaatgaggaagctgcagaatgggatagagtgcatccagtgcatgc |
| SEQ ID NO:74 | pMDLg/pRRE-D64V-MA-PP7x1 Oligo Insert Amino Acid Sequence | MGARASVLSGGELDRWEKIRLRPGGKKKYKLKHIVWASRELERGGGGGMASNFTQFVLVDSKTIVLSVGEATRTLTEIQSTADRQIFEEKVGPLVGRLRLTASLRQNGAKTAYRVNLKLDQADVVDSGLPKVRYTQVWSHDVTIVANSTEASRKSLYDLTKSLVATSQVEDLVVNLVPLGRNQVSQNYPIVQNIQGQMVHQAISPRTLNAWVKVVEEKAFSPEVIPMFSALSEGATPQDLNTMLNTVGGHQAAMQMLKETINEEAAEWDRVHPVH |
| SEQ ID NO:75 | pMDLg/pRRE-D64V-MA-PP7x2 DNA Oligo Insert | cacgtgagatctgaattcgagatctgccgccgccatgggtgcgagagcgtcagtattaagcgggggagaattagatcgatgggaaaaaattcggttaaggccagggggaaagaaaaaatataaattaaaacatatagtatgggcaagcagggagctagaacgaggaggtggtggaggaatggcttctaactttactcagttcgttctcgtcgactccaaaacaatagtcctctccgtaggggaggcaacacggactttgaccgaaatccagtcaaccgctgaccgacaaatctttgaagagaaagtagggcctcttgtgggccgactgcgcttgactgcaagcttgcgacaaaacggcgcaaagactgcctatagggtcaaccttaaactcgaccaagccgacgtggtcgatagcggtctccctaaggttcggtatacgcaggtctggagtcatgacgtaacaatcgtagcaaacagcacagaagcctcccgaaaaagcctctacgatctgacgaaatccttggtggctacgtcacaggtggaagacctcgttgtcaaccttgtacctctgggtcgagcggatccgctcgcatcaaaaactattgtgctctccgtgggagaagccacccgcacgcttaccgaaattcaatcaacggcagacagacaaatctttgaggagaaagtaggtccgttggtgggtcggttgcgcttgaccgcaagcctccgccaaaacggagcgaaaaccgcataccgcgtaaacttgaagctggaccaagccgatgttgttgactccggcttgcccaaagtgcgatatactcaggtctggtctcatgatgtcacaatcgtcgctaattccactgaggctagtcgcaaaagtctgtatgacttgacaaagtccttggtagccacgtcacaggtggaagatttggtggtgaacctcgttccactgggaagaaaccaggtcagccaaaattaccctatagtgcagaacatccaggggcaaatggtacatcaggccatatcacctagaactttaaatgcatgggtaaaagtagtagaagagaaggctttcagcccagaagtgatacccatgttttcagcattatcagaaggagccaccccacaagatttaaacaccatgctaaacacagtggggggacatcaagcagccatgcaaatgttaaaagagaccatcaatgaggaagctgcagaatgggatagagtgcatccagtgcatgc |
| SEQ ID NO:76 | pMDLg/pRRE-D64V-MA-PP7x2 Oligo Insert Amino Acid Sequence | MGARASVLSGGELDRWEKIRLRPGGKKKYKLKHIVWASRELERGGGGGMASNFTQFVLVDSKTIVLSVGEATRTLTEIQSTADRQIFEEKVGPLVGRLRLTASLRQNGAKTAYRVNLKLDQADVVDSGLPKVRYTQVWSHDVTIVANSTEASRKSLYDLTKSLVATSQVEDLVVNLVPLGRADPLASKTIVLSVGEATRTLTEIQSTADRQIFEEKVGPLVGRLRLTASLRQNGAKTAYRVNLKLDQADVVDSGLPKVRYTQVWSHDVTIVANSTEASRKSLYDLTKSLVATSQVEDLVVNLVPLGRNQVSQNYPIVQNIQGQMVHQAISPRTLNAWVKVVEEKAFSPEVIPMFSALSEGATPQDLNTMLNTVGGHQAAMQMLKETINEEAAEWDRVHPVH |
| SEQ ID NO:81 | DNA oligo cloned into Plasmid No. 30 (pSaCas9^1PP7^-HBB-sgRNA1^3’ PP7^) encoding the U6 promoter, HBB sgRNA1 and PP7 aptamer | GGTACCGAGGGCCTATTTCCCATGATTCCTTCATATTTGCATATACGATACAAGGCTGTTAGAGAGATAATTGGAATTAATTTGACTGTAAACACAAAGATATTAGTACAAAATACGTGACGTAGAAAGTAATAATTTCTTGGGTAGTTTGCAGTTTTAAAATTATGTTTTAAAATGGACTATCATATGCTTACCGTAACTTGAAAGTATTTCGATTTCTTGGCTTTATATATCTTGTGGAAAGGACGAAACACCGAGTAACGGCAGACTTCTCCACGTTCTAGTACTCTGGAAACAGAATCTACTAGAACAAGGCAAAATGCCGTGTTTATCTCGTCAACTTGTTGGCGAGATGGAGCAGACGATATGGCGTCGCTCCTTTTTTTGCGGCCGC |
| SEQ ID NO:82 | DNA oligo cloned into Plasmid No. 32 (pAAV-HBB-sgRNA2) encoding the human HBB target template sequence and the U6 driven HBB sgRNA2 expression cassette | gcggccgcttgtgtaatcgtagtttcagagtgttagagctgaaaggaagaagtaggagaaacatgcaaagtaaaagtataacactttccttactaaaccgacatgggtttccaggtaggggcaggattcaggatgactgacagggcccttagggaacactgagaccctacgctgacctcataaatgcttgctacctttgctgttttaattacatcttttaatagcaggaagcagaactctgcacttcaaaagtttttcctcacctgaggagttaatttagtacaaggggaaaaagtacagggggatgggagaaaggcgatcacgttgggaagctatagagaaagaagagtaaattttagtaaaggaggtttaaacaaacaaaatataaagagaaataggaacttgaatcaaggaaatgattttaaaacgcagtattcttagtggactagaggaaaaaaataatctgagccaagtagaagaccttttcccctcctacccctactttctaagtcacagaggctttttgttcccccagacactcttgcagattagtccaggcagaaacagttagatgtccccagttaacctcctatttgacaccactgattaccccattgatagtcacactttgggttgtaagtgactttttatttatttgtatttttgactgcattaagaggtctctagttttttatctcttgtttcccaaaacctaataagtaactaatgcacagagcacattgatttgtatttattctatttttagacataatttattagcatgcatgagcaaattaagaaaaacaacaacaaatgaatgcatatatatgtatatgtatgtgtgtatatatacacacatatatatatatattttttcttttcttaccagaaggttttaatccaaataaggagaagatatgcttagaaccgaggtagagttttcatccattctgtcctgtaagtattttgcatattctggagacgcaggaagagatccatctacatatcccaaagctgaattatggtagacaaaactcttccacttttagtgcatcaacttcttatttgtgtaataagaaaattgggaaaacgatcttcaatatgcttaccaagctgtgattccaaatattacgtaaatacacttgcaaaggaggatgtttttagtagcaatttgtactgatggtatggggccaagagatatatcttagagggagggctgagggtttgaagtccaactcctaagccagtgccagaagagccaaggacaggtacggctgtcatcacttagacctcaccctgtggagccacaccctagggttggccaatctactcccaggagcagggagggcaggagccagggctgggcataaaagtcagggcagagccatctattgcttacatttgcttctgacacaactgtgttcactagcaacctcaaacagacaccatggtgcatctgactcctgaggagaaaagcgctgtgacagctctctggggaaaagtcaatgtcgacgaagttggtggtgaggccctgggcaggttggtatcaaggttacaagacaggtttaaggagaccaatagaaactgggcatgtggagacagagaagactcttgggtttctgataggcactgactctctctgcctattggtctattttcccacccttaggctgctggtggtctacccttggacccagaggttctttgagtcctttggggatctgtccactcctgatgctgttatgggcaaccctaaggtgaaggctcatggcaagaaagtgctcggtgcctttagtgatggcctggctcacctggacaacctcaagggcacctttgccacactgagtgagctgcactgtgacaagctgcacgtggatcctgagaacttcagggtgagtctatgggacgcttgatgttttctttccccttcttttctatggttaagttcatgtcataggaaggggataagtaacagggtacagtttagaatgggaaacagacgaatgattgcatcagtgtggaagtctcaggatcgttttagtttcttttatttgctgttcataacaattgttttcttttgtttaattcttgctttctttttttttcttctccgcaatttttactattatacttaatgccttaacattgtgtataacaaaaggaaatatctctgagatacattaagtaacttaaaaaaaaactttacacagtctgcctagtacattactatttggaatatatgtgtgcttatttgcatattcataatctccctactttattttcttttatttttaattgatacataatcattatacatatttatgggttaaagtgtaatgttttaatatgtgtacacatattgaccaaatcagggtaattttgcatttgtaattttaaaaaatgctttcttcttttaatatacttttttgtttatcttatttctaatactttccctaatctctttctttcagggcaataatgatacaatgtatcatgcctctttgcaccattctaaagaataacagtgataatttctgggttaaggcaatagcaatatctctgcatataaatatttctgcatataaattgtaactgatgtaagaggtttcatattgctaatagcagctacaatccagctaccattctgcttttattttatggttgggataaggctggattattctgagtccaagctaggcccttttgctaatcatgttcatacctcttatcttcctcccacagctcctgggcaacgtgctggtctgtgtgctggcccatcactttggcaaagaattcaccccaccagtgcaggctgcctatcagaaagtggtggctggtgtggctaatgccctggcccacaagtatcactaagctcgctttcttgctgtccaatttctattaaaggttcctttgttccctaagtccaactactaaactgggggatattatgaagggccttgagcatctggattctgcctaataaaaaacatttattttcattgcaatgatctcgagggcctatttcccatgattccttcatatttgcatatacgatacaaggctgttagagagataattggaattaatttgactgtaaacacaaagatattagtacaaaatacgtgacgtagaaagtaataatttcttgggtagtttgcagttttaaaattatgttttaaaatggactatcatatgcttaccgtaacttgaaagtatttcgatttcttggctttatatatcttgtggaaaggacgaaacaccgccctgtggggcaaggtgaacgttttagtactctggaaacagaatctactaaaacaaggcaaaatgccgtgtttatctcgtcaacttgttggcgagatttttctagagcggccgc |
| SEQ ID NO:83 | DNA oligo cloned into Plasmid No. 36 (pSaCas9^1xms2^-2x3’UTR) encoding two copies of the human HBB 3’ UTR | ggatcctaagctcgctttcttgctgtccaatttctattaaaggttcctttgttccctaagtccaactactaaactgggggatattatgaagggccttgagcatctggattctgcctaataaaaaacatttattttcattgctagctcgctttcttgctgtccaatttctattaaaggttcctttgttccctaagtccaactactaaactgggggatattatgaagggccttgagcatctggattctgcctaataaaaaacatttattttcattgc |
| SEQ ID NO:84 | DNA oligo cloned into Plasmid No. 41 (pSaCas9^1xMS2^-2x3'UTR-HBB sgRNA^3’PP7^ 1x3'UTR) encoding HBB sgRNA1 with one PP7 aptamer and one copy of HBB 3’ UTR downstream of U6 promoter | CGGCCGAGGGCCTATTTCCCATGATTCCTTCATATTTGCATATACGATACAAGGCTGTTAGAGAGATAATTGGAATTAATTTGACTGTAAACACAAAGATATTAGTACAAAATACGTGACGTAGAAAGTAATAATTTCTTGGGTAGTTTGCAGTTTTAAAATTATGTTTTAAAATGGACTATCATATGCTTACCGTAACTTGAAAGTATTTCGATTTCTTGGCTTTATATATCTTGTGGAAAGGACGAAACACCGAGTAACGGCAGACTTCTCCACGTTCTAGTACTCTGGAAACAGAATCTACTAGAACAAGGCAAAATGCCGTGTTTATCTCGTCAACTTGTTGGCGAGATATCGGAGCAGACGATATGGCGTCGCTCCAGCGCTCGCTTTCTTGCTGTCCAATTTCTATTAAAGGTTCCTTTGTTCCCTAAGTCCAACTACTAAACTGGGGGATATTATGAAGGGCCTTGAGCATCTGGATTCTGCCTAATAAAAAACATTTATTTTCATTGCTTTTTTTGCGGCCG |
| SEQ ID NO:85 | DNA oligo cloned into Plasmid No. 42 (pSaCas9^1xMS2^-2x3'UTR-HBB sgRNA^3’PP7^ 2x3'UTR) encoding one PP7 aptamer sequence followed by two HBB 3’ UTR sequences | GATATCGGAGCAGACGATATGGCGTCGCTCCAGCGCTCGCTTTCTTGCTGTCCAATTTCTATTAAAGGTTCCTTTGTTCCCTAAGTCCAACTACTAAACTGGGGGATATTATGAAGGGCCTTGAGCATCTGGATTCTGCCTAATAAAAAACATTTATTTTCATTGCGCTCGCTTTCTTGCTGTCCAATTTCTATTAAAGGTTCCTTTGTTCCCTAAGTCCAACTACTAAACTGGGGGATATTATGAAGGGCCTTGAGCATCTGGATTCTGCCTAATAAAAAACATTTATTTTCATTGCTTTTTTTGCGGCCGC |
| SEQ ID NO:86 | Oligo HBA-5’-F | Ctggctaactaccggctcttctggtccccacagactcagagagaaccggtgccaccatgg |
| SEQ ID NO:87 | Oligo HBA-5’-R | ccatggtggcaccggttctctctgagtctgtggggaccagaagagccggtagttagccaG |
| SEQ ID NO:88 | Oligo sp-loop1F | AAAAAAGAAAAAGCTTTAGAAAACATGAGGATCACCCATGTCTGCAGGTCGACTCTAGAATTCCTAGAGCTCG |
| SEQ ID NO:89 | Oligo sp-loop1R | CGAGCTCTAGGAATTCTAGAGTCGACCTGCAGACATGGGTGATCCTCATGTTTTCTAAAGCTTTTTCTTTTTT |
| SEQ ID NO:90 | Il2RG-sp-g1F1 | ACCGGCGCTTGCTCTTCATTCCCT |
| SEQ ID NO:91 | Il2RG-sp-g1R | AAACAGGGAATGAAGAGCAAGCGC |
| SEQ ID NO:92 | MS2-F1 oligo | ACTTGTTGGCGAGATATCTAGAAAACATGAGGATCACCCATGTCTGCAGAGCGCTCGCTTTCTTGCT |
| SEQ ID NO:93 | MS2-R1 oligo | AGCAAGAAAGCGAGCGCTCTGCAGACATGGGTGATCCTCATGTTTTCTAGATATCTCGCCAACAAGT |
| SEQ ID NO:94 | Human beta hemoglobin DNA sequence (The “T” in the underlined codon is the mutated nucleotide. The underlined codon encodes the sixth amino acid of the β globin protein.) | ACTCCTG**T**GGAGAAGTCTGCCGTTACT |
| SEQ ID NO:95 | 119 bp DNA Insertion in human beta hemoglobin (HBB) gene in EGFP reporter cells as described in Javidi-Parsijani, P. et al., *PLoS One* 2017; **12**(5): e0177444 | GAACCCAGGTTCCTGACACAGACAGACTACACCCAGGGAATGAAGAGCAAGCGCCATACTCCTGTGGAGAAGTCTGCCGTTACTGCCCTGTGGGGCAAGGTGAACGTGGATTGGCTAGC |
| SEQ ID NO:96 | HBB-1849F | CGATCACGTTGGGAAGCTATAGAG |
| SEQ ID NO:97 | HBB-5277R | AACATCCTGAGGAAGAATGGGAC |
| SEQ ID NO:98 | Reporter-mut-F1 | TCGTCGGCAGCGTCAGATGTGTATAAGAGACAGTtccatttcaggtgtcgtgag |
| SEQ ID NO:99 | Reporter-mut-F2 | TCGTCGGCAGCGTCAGATGTGTATAAGAGACAGATtccatttcaggtgtcgtgag |
| SEQ ID NO:100 | Reporter-mut-F3 | TCGTCGGCAGCGTCAGATGTGTATAAGAGACAGGATtccatttcaggtgtcgtgag |
| SEQ ID NO:101 | Reporter-mut-F4 | TCGTCGGCAGCGTCAGATGTGTATAAGAGACAGCGATtccatttcaggtgtcgtgag |
| SEQ ID NO:102 | Reporter-mut-R1 | GTCTCGTGGGCTCGGAGATGTGTATAAGAGACAGTGAACTTCAGGGTCAGCTTGC |
| SEQ ID  NO: 103 | Synthetic fragment containing *IL2RG sgRNA2*- expression cassette and *IL2RG* template for homologous recombination.  Letters in green: 5’ arm;  Underlined: IL2RG cDNA;  Letters in blue: 3’ arm;  Letters in red: IL2RG sgRNA2 and sgRNA4 expression cassettes. | GCGGCCgcTCTCGAACTCCTCAAGCAATCCACCTGCCTTGGCCTCCCAAAGTGCTGGGATTGCAGGCGTGAGTCACTGCACCCAGCCGAGAGAATAAATTTCTGTTGGTTTAAGCCACTCAGTTTGGGGATAACTTATGGCAGCCCTAGCAAACTAATACATACTAAAGATACATACTAAATACTAAGCTGGGCCATATAGTCCAGTTTTCCTGAGACTCCCAGGCAAGTGCTGTTTTTCTTTGCTTAATATCCTACACCACTTTCTGTCTGGTAAAATTACACTCATTCTTTAAGATGCCACTGAAATAGCACCTCTTCAGCACAGCCTTCACTAAACTATCCCCCTCTCCATCTTGGTAAATTTAGTTACTTCCTCTTCTGTGCTCACATACTTTGTAGTATCTCTACATTTATGCTATAGGACTTGTTACACTATGTTGTATTACTTGTTTATGTCTTCCCCACTTTTCTGTGAGTGTCTAGAAATATGAGGATGTCTTGTTGGTCTATTTCCAGAACATAAGCACAGTGCCTGGCACATATTAAAAACGTAATAAATGTTTGCTGAATAAATAGTTTCTGTAAGTGGCTTCTCCAATCACCTCTGTGTTTTCGGGGAAGGTAAAACTGGCAACAGGATGAAGAATGGATTAGAGAGCAGAGGGCCTTTAGAAAGGGAGGCCAGTTGATGGAGTCTAGATAGAATCATGACTAGAGCTAATGAAAGACTGATTTAGCAGAGTGGCTGTGGTAATGGAAAGGAGGAAACCGTTGGGAGAAACACCACAGAAGCAGAGTGGGTTATATTCTCTGGGTGAGAGAGGGGGAGAAATTGAAGCTGATTCTGAGGTTTCAAGTCTGGGTGACTGAGAGGGTGACGATACCATTGACTGAGGTGGGGAAGGCAGGAAGAGAAGCAGAGTTGGGGGAAGATGGGAAGCTTGAAGCTAGTATTGTTGTTCCTCCATTTCTAGAATATTTTTGTATTATAAGTCACACTTCCTCGCCAGTCTCAACAGGGACCCAGCTCAGGCAGCAGCTAAGGGTGGGTATTCTGGTTTGGATTAGATCAGAGGAAAGACAGCTGTATATGTGCCCACAGGAGCCAAGACGGTATTTTCCATCCTCCCAAAACAGTAGAGCTTTGACAGAGATTTAAGGGTGACCAAGTCAAGGAAGAGGCATGGCATAGAACGGTGATGTCGGGGGTGGGGGTTCAGAACTTCCATTATAGAAGGTAATGATTTAGAGGAGAAGGTGGTTGAGAATGGTGCTAGTGGTAGTGAACAGATCCTTCCCAGGATCTAGGTGGGCTGAGGATTTTTGAGTCTGTGACACTATTGTATATCCAGCTTTAGTTTCTGTTTACCACCTTACAGCAGCACCTAATCTCCTAGAGGACTTAGCCCGTGTCACACAGCACATATTTGCCACACCCTCTGTAAAGCCCTGGTTTATAAGGTTCTTTCCACCGGAAGCTATGACAGAGGAAACGTGTGGGTGGGGAGGGGTAGTGGGTGAGGGACCCAGGTTCCTGccAccatgCtCaaAccTtcCCtGccTttTacCAGcTtGCtGttTctCcagctCccTctCctCggCgtCggActCaaTacAacTatCctGacAccTaaCggAaaCgaGgaTacAacCgcCgaCttTttTctCacAacCatgccTacAgaTAGcTtGTCCgtGAGcacCctCccTctGccTgaAgtGcagtgCttCgtCttTaaCgtGgaAtaTatgaaCtgTacCtggaaTTCcTCcAGCgaAccTcagccAacAaaTctGacActCcaCtaCtggtaTaaAaaTAGCgaCaaCgaCaaGgtGcagaaAtgTTCccaTtaCTTGttTAGCgaGgaGatTacCAGCggAtgCcagCtCcaGaaGaaAgaAatTcaTctGtaTcaGacCttCgtGgtGcagctGcaggaTccTAgAgaGccTagAagGcaggcTacCcagatgTTGaaGctCcagaaCctCgtCatTccTtgggcCccTgaAaaTctGacCTtGcaTaaGctCTCCgaGAGccagctGgaGctCaaTtggaaTaacagGttTCtgaaTcaTtgCCtggaAcaTCTCgtCcaAtaTAGAacCgaTtgggaTcaTTCctggacCgaGcaGAGCgtCgaCtaCCgGcaCaaAttTAGcCtgccAagCgtCgaCggAcagaaGAgAtaTacCttCAgAgtGAgATCcAgAttCaaTccTctGtgCggCTCCgcAcagcaCtggTCCgaGtggTCccaTccTatTcaTtggggATCcaaCacCAGCaaGgaAaaCccAttTctgttCgcTCTggaGgcTgtCgtGatTAGCgtGggAAGcatgggCCtgatCatTTCcTTGctGtgCgtTtaCttTtggctggaGAgAacCatgccTAgGatCccTacActCaaAaaTctGgaAgaCTTGgtGacAgaGtaTcaTggAaaTttCAGCgcTtggTCCggAgtCAGCaaAggCctCgcCgaAagCctCcagccTgaTtaTagCgaGAgGctGtgTctGgtGagCgaAatCccTccTaaGggCggAgcTctGggCgaAggAccAggCgCTAGCCCTTGTAATCAGCACTCCCCTTATTGGGCTCCTCCTTGCTATACATTGAAACCAGAGACATAAGGGAACCCAGGAGACAGGCCACACAGATGCTAAAACTGCAGAATCTGGGTAATTTGGAAAGAAAGGGTCAAGAGACCAGGGATACTGTGGGACATTGGAGTCTACAGAGTAGTGTTCTTTTATCATAAGGGTACATGGGCAGAAAAGAGGAGGTAGGGGATCATGATGGGAAGGGAGGAGGTATTAGGGGCACTACCTTCAGGATCCTGACTTGTCTAGGCCAGGGGAATGACCACATATGCACACATATCTCCAGTGATCCCCTGGGCTCCAGAGAACCTAACACTTCACAAACTGAGTGAATCCCAGCTAGAACTGAACTGGAACAACAGATTCTTGAACCACTGTTTGGAGCACTTGGTGCAGTACCGGACTGACTGGGACCACAGCTGGACTGTGAGTGACTAGGGACGTGAATGTAGCAGCTAAGGCCAAGAAAGTAGGGCTAAAGGATTCAACCAGACAGATAGAAGGACCTAATATCAAGCTCCTGTTCTCTGCCTCCCAGCTTCTCTGCTCACCCCCTACCCTCCCTCCTCCAACTCCTTTCCTCGAGGGCCTATTTCCCATGATTCCTTCATATTTGCATATACGATACAAGGCTGTTAGAGAGATAATTGGAATTAATTTGACTGTAAACACAAAGATATTAGTACAAAATACGTGACGTAGAAAGTAATAATTTCTTGGGTAGTTTGCAGTTTTAAAATTATGTTTTAAAATGGACTATCATATGCTTACCGTAACTTGAAAGTATTTCGATTTCTTGGCTTTATATATCTTGTGGAAAGGACGAAACACCGTGGCCTGTCTCCTGGGTTCCCGTTTTAGTACTCTGGAAACAGAATCTACTAAAACAAGGCAAAATGCCGTGTTTATCTCGTCAACTTGTTGGCGAGATTTTTTGTTTTAGAGCTAGAAATAGCAAGTTAAAATAAGGCTAGTCCGTTTTTAGCGCGTGCGCCAATTCTGCAGACAAATGAGGGCCTATTTCCCATGATTCCTTCATATTTGCATATACGATACAAGGCTGTTAGAGAGATAATTGGAATTAATTTGACTGTAAACACAAAGATATTAGTACAAAATACGTGACGTAGAAAGTAATAATTTCTTGGGTAGTTTGCAGTTTTAAAATTATGTTTTAAAATGGACTATCATATGCTTACCGTAACTTGAAAGTATTTCGATTTCTTGGCTTTATATATCTTGTGGAAAGGACGAAACACCGGACACAGACAGACTACACCCAGTTTTAGTACTCTGGAAACAGAATCTACTAAAACAAGGCAAAATGCCGTGTTTATCTCGTCAACTTGTTGGCGAGATTTTTggtAccggtGCGGCCGC |
| Seq ID 104 | HBB sgRNA1 expression cassette and the wild type template.  Green letters: HBB 5’ arm.  Blue letters: HBB 3’ arm.  Red letters: HBB sgRNA1 expression cassette. | GCggccgcttgtgtaatcgtagtttcagagtgttagagctgaaaggaagaagtaggagaaacatgcaaagtaaaagtataacactttccttactaaaccgacatgggtttccaggtaggggcaggattcaggatgactgacagggcccttagggaacactgagaccctacgctgacctcataaatgcttgctacctttgctgttttaattacatcttttaatagcaggaagcagaactctgcacttcaaaagtttttcctcacctgaggagttaatttagtacaaggggaaaaagtacagggggatgggagaaaggcgatcacgttgggaagctatagagaaagaagagtaaattttagtaaaggaggtttaaacaaacaaaatataaagagaaataggaacttgaatcaaggaaatgattttaaaacgcagtattcttagtggactagaggaaaaaaataatctgagccaagtagaagaccttttcccctcctacccctactttctaagtcacagaggctttttgttcccccagacactcttgcagattagtccaggcagaaacagttagatgtccccagttaacctcctatttgacaccactgattaccccattgatagtcacactttgggttgtaagtgactttttatttatttgtatttttgactgcattaagaggtctctagttttttatctcttgtttcccaaaacctaataagtaactaatgcacagagcacattgatttgtatttattctatttttagacataatttattagcatgcatgagcaaattaagaaaaacaacaacaaatgaatgcatatatatgtatatgtatgtgtgtatatatacacacatatatatatatattttttcttttcttaccagaaggttttaatccaaataaggagaagatatgcttagaaccgaggtagagttttcatccattctgtcctgtaagtattttgcatattctggagacgcaggaagagatccatctacatatcccaaagctgaattatggtagacaaaactcttccacttttagtgcatcaacttcttatttgtgtaataagaaaattgggaaaacgatcttcaatatgcttaccaagctgtgattccaaatattacgtaaatacacttgcaaaggaggatgtttttagtagcaatttgtactgatggtatggggccaagagatatatcttagagggagggctgagggtttgaagtccaactcctaagccagtgccagaagagccaaggacaggtacggctgtcatcacttagacctcaccctgtggagccacaccctagggttggccaatctactcccaggagcagggagggcaggagccagggctgggcataaaagtcagggcagagccatctattgcttacatttgcttctgacacaactgtgttcactagcaacctcaaacagacaccatggtgcatctgactcctgaggagaaaagcgctgtgacagctctctggggaaaagtcaatgtcgacgaagttggtggtgaggccctgggcaggttggtatcaaggttacaagacaggtttaaggagaccaatagaaactgggcatgtggagacagagaagactcttgggtttctgataggcactgactctctctgcctattggtctattttcccacccttaggctgctggtggtctacccttggacccagaggttctttgagtcctttggggatctgtccactcctgatgctgttatgggcaaccctaaggtgaaggctcatggcaagaaagtgctcggtgcctttagtgatggcctggctcacctggacaacctcaagggcacctttgccacactgagtgagctgcactgtgacaagctgcacgtggatcctgagaacttcagggtgagtctatgggacgcttgatgttttctttccccttcttttctatggttaagttcatgtcataggaaggggataagtaacagggtacagtttagaatgggaaacagacgaatgattgcatcagtgtggaagtctcaggatcgttttagtttcttttatttgctgttcataacaattgttttcttttgtttaattcttgctttctttttttttcttctccgcaatttttactattatacttaatgccttaacattgtgtataacaaaaggaaatatctctgagatacattaagtaacttaaaaaaaaactttacacagtctgcctagtacattactatttggaatatatgtgtgcttatttgcatattcataatctccctactttattttcttttatttttaattgatacataatcattatacatatttatgggttaaagtgtaatgttttaatatgtgtacacatattgaccaaatcagggtaattttgcatttgtaattttaaaaaatgctttcttcttttaatatacttttttgtttatcttatttctaatactttccctaatctctttctttcagggcaataatgatacaatgtatcatgcctctttgcaccattctaaagaataacagtgataatttctgggttaaggcaatagcaatatctctgcatataaatatttctgcatataaattgtaactgatgtaagaggtttcatattgctaatagcagctacaatccagctaccattctgcttttattttatggttgggataaggctggattattctgagtccaagctaggcccttttgctaatcatgttcatacctcttatcttcctcccacagctcctgggcaacgtgctggtctgtgtgctggcccatcactttggcaaagaattcaccccaccagtgcaggctgcctatcagaaagtggtggctggtgtggctaatgccctggcccacaagtatcactaagctcgctttcttgctgtccaatttctattaaaggttcctttgttccctaagtccaactactaaactgggggatattatgaagggccttgagcatctggattctgcctaataaaaaacatttattttcattgcaatgatctcgagggcctatttcccatgattccttcatatttgcatatacgatacaaggctgttagagagataattggaattaatttgactgtaaacacaaagatattagtacaaaatacgtgacgtagaaagtaataatttcttgggtagtttgcagttttaaaattatgttttaaaatggactatcatatgcttaccgtaacttgaaagtatttcgatttcttggctttatatatcttgtggaaaggacgaaacaccGAGTAACGGCAGACTTCTCCACgttttagtactctggaaacagaatctactaaaacaaggcaaaatgccgtgtttatctcgtcaacttgttggcgagatttttgcGGCCGC |
|  | HBB-F2: | GGGCAGAGCCATCTATTGCTTA |
|  | HBB-R3: | TGGGAAAATAGACCAATAGGCAGAG |
|  | SaCas9-2576F | AAACCGGGAACTACCTGACC |
|  | SaCas9- 2713R | TCACGACCTTGTTTCTGCTG |
|  | SgRNA-F1 | GAGTAACGGCAGACTTCTCCA |
|  | sgRNA-R1 | CGGCATTTTGCCTTGTTTAAG |
|  | sgRNA-R2 | CGCCAACAAGTTGACGAGAT |
|  | sgRNA-R3 | GATAAACACGGCATTTTGCCTTG |
|  | EGFP-RT-F | CAGTGCTTCAGCCGCTACCC |
|  | EGFP-RT-R | AGCTCGATGCGGTTCACCAG |
|  | HBB-mut-F1 | TCGTCGGCAGCGTCAGATGTGTATAAGAGACAGTGTTCACTAGCAACCTCAAACAG |
|  | HBB-mut-F2 | TCGTCGGCAGCGTCAGATGTGTATAAGAGACAGATGTTCACTAGCAACCTCAAACAG |
|  | HBB-mut-F3 | TCGTCGGCAGCGTCAGATGTGTATAAGAGACAGGATGTTCACTAGCAACCTCAAACAG |
|  | HBB-mut-F4 | TCGTCGGCAGCGTCAGATGTGTATAAGAGACAGCGATGTTCACTAGCAACCTCAAACAG |
|  | HBB-mut-F5 | TCGTCGGCAGCGTCAGATGTGTATAAGAGACAGTCGATGTTCACTAGCAACCTCAAACAG |
|  | HBB-mut-F6 | TCGTCGGCAGCGTCAGATGTGTATAAGAGACAGATCGATGTTCACTAGCAACCTCAAACAG |
|  | HBB-mut-R1 | GTCTCGTGGGCTCGGAGATGTGTATAAGAGACAGTCCAATAGGCAGAGAGAGTCAGTG |
|  | HBB_off-F1 | TCGTCGGCAGCGTCAGATGTGTATAAGAGACAGTtctgtcacccaggctagagt |
|  | HBB_off-R1 | GTCTCGTGGGCTCGGAGATGTGTATAAGAGACAGTggctgaggtgggagaatcac |
|  | HBB_off-F2 | TCGTCGGCAGCGTCAGATGTGTATAAGAGACAGTatttctcctctgcactgccc |
|  | HBB_off-R2 | GTCTCGTGGGCTCGGAGATGTGTATAAGAGACAGTggagcctcggcctagatttc |
|  | HBB_off-F3 | TCGTCGGCAGCGTCAGATGTGTATAAGAGACAGTTGATCCCTTCCACCAATGTC |
|  | HBB_off-R3 | GTCTCGTGGGCTCGGAGATGTGTATAAGAGACAGTCACGGCTAGGAATAGCAAGG |
|  | HBB_off-F4 | TCGTCGGCAGCGTCAGATGTGTATAAGAGACAGTAAAGGAAATTCCATCAGACTAACG |
|  | HBB_off-R4 | GTCTCGTGGGCTCGGAGATGTGTATAAGAGACAGTCTTTGAAAATGCCGTCCATC |
|  | HBB_off-F5 | TCGTCGGCAGCGTCAGATGTGTATAAGAGACAGTAAGACAAAAGCAGAAGGTAAGC |
|  | HBB_off-R5 | GTCTCGTGGGCTCGGAGATGTGTATAAGAGACAGTAAGTTGAAGATAGGACCCCACTC |
|  | HBB_off-F6 | TCGTCGGCAGCGTCAGATGTGTATAAGAGACAGTCCTATAAAGGGAAGCCCATC |
|  | HBB_off-R6 | GTCTCGTGGGCTCGGAGATGTGTATAAGAGACAGTGGCAACAAGAGCAAAACTCC |
|  | HBB_off-F7 | TCGTCGGCAGCGTCAGATGTGTATAAGAGACAGTCCAGAAAGGGAAAACTTGCAC |
|  | HBB_off-R7 | GTCTCGTGGGCTCGGAGATGTGTATAAGAGACAGTCCACTGACCCAATCTTTTCC |
|  | HBB_off-F8 | TCGTCGGCAGCGTCAGATGTGTATAAGAGACAGTCTGCTCTTTGCCTGTTGGAG |
|  | HBB_off-R8 | GTCTCGTGGGCTCGGAGATGTGTATAAGAGACAGTGCTAAAGCTGGAAGGCTGTG |
|  | IL2RG-1029F: | ATGCCCTCTGTAGTGGGTTG |
|  | IL2RG-3301R | GGCAGCTGCAGGAATAAGAG |
|  | IL2RG-mut-F1 | TCGTCGGCAGCGTCAGATGTGTATAAGAGACAGTGAAGCTATGACAGAGGAAACG |
|  | IL2RG-mut-R4 | GTCTCGTGGGCTCGGAGATGTGTATAAGAGACAGTGCAGCTGCAGGAATAAGAGG |

Note: Sequence numbers are non-continuous to keep the same identity for each in the lab.
