# Supplementary material for: Delivering SaCas9 mRNA by lentivirus-like bionanoparticles for transient expression and efficient genome editing
Source: Nucleic Acids Res. 2019 Feb 13;47(8):e44. doi: 10.1093/nar/gkz093 (PMC6486560; doi:10.1093/nar/gkz093)
Supplement: Supplementary Data [file nar_47_8_e44_s1.zip › Supplementry table 1.docx]

**Supplementary Table 1. Plasmids made for the study^a^**

| No. | Name | Purpose | Generation strategy |
| --- | --- | --- | --- |
| 1 | pCDNA3.1/MS2x2-VPR | Expressing MS2x2-VPR fusion protein | Synthesized DNA coding for MS2x2-VPR (Genscript, Piscataway, NJ) was inserted between the NheI and NotI sites of pCDNA3.1(+) (Genscript). The inserted DNA sequence is SEQ ID NO:61 and the corresponding amino acid sequence is SEQ ID NO:62. |
| 2 | pCDNA3.1/NEF-MS2x2 | Expressing NEF-MS2x2 fusion protein. Three AA of NEF were mutated: Gly^3^, Val^153^ and Gly^177^ were changed to Cyc, Leu and Glu, respectively. | Synthesized DNA coding for NEF-MS2x2 was inserted between the NheI and NotI sites of pCDNA3.1(+) (Genscript). The inserted DNA sequence is SEQ ID NO:63 and the corresponding amino acid sequence is SEQ ID NO:64. |
| **Packaging Plasmids** | | | |
| 3 | pMDLg/pRRE-D64V | Third generation lentivirus packaging plasmid for generating integration defective lentivirus. | AflII-AgeI fragment of pMDLg/pRRE was replaced by the AflII-AgeI fragment of psPAX2-D64V. |
| 4 | pMDLg/pRRE-D64V-NC-MS2x1 | Third generation lentivirus packaging plasmid with MCP inserted after NC ZF2. | AvrII-SbfI fragment of pMDLg/pRRE-D64V was replaced by a synthetic AvrII-SbfI DNA fragment encoding a modified NC sequence in which one copy of MCP coding DNA (stop codons removed) was inserted in frame after the last codon of the NC zinc finger 2 domain. The inserted DNA sequence is SEQ ID NO:65 and the corresponding amino acid sequence is SEQ ID NO:66. |
| 5 | pMDLg/pRRE-D64V-NC-MS2x2 | Third generation lentivirus packaging plasmid with two copies of MCP inserted after NC ZF2. | AvrII-SbfI fragment of pMDLg/pRRE-D64V was replaced by a synthetic AvrII-SbfI DNA fragment encoding a modified NC sequence in which two copies of MCP coding DNA (stop codons removed) were inserted in frame after the last codon of NC zinc finger 2 domain. The inserted DNA sequence is SEQ ID NO:67 and the corresponding amino acid sequence is SEQ ID NO:68. |
| 6 | pMDLg/pRRE-D64V-NC-PP7x1 | Third generation lentivirus packaging plasmid with one copy of PP7 coat protein (PCP) inserted after NC ZF2. | AvrII-SbfI fragment of pMDLg/pRRE-D64V was replaced by a synthetic AvrII-SbfI DNA fragment encoding a modified NC sequence in which one copy of PCP coding DNA (stop codons removed) was inserted in frame after the last codon of NC zinc finger 2 domain. The inserted DNA sequence is SEQ ID NO:69 and the corresponding amino acid sequence is SEQ ID NO:70. |
| 8 | pMDLg/pRRE-D64V-MA-MS2x2 | Third generation lentivirus packaging plasmid with two copies of MCP replacing AA 44-132 of MA. | The PmlI-SphI fragment of pMDLg/pRRE-D64V was replaced by a synthetic PmlI-SphI DNA fragment encoding a modified MA in which two copies of MCP coding DNA (stop codons removed) replaced DNA coding for AA 44-132 of MA. The inserted DNA sequence is SEQ ID NO:71 and the corresponding amino acid sequence is SEQ ID NO:72. |
| 9 | pMDLg/pRRE-D64V-MA-PP7x1 | Third generation lentivirus packaging plasmid with one copy of PCP replacing AA 44-132 of MA. | The PmlI-SphI fragment of pMDLg/pRRE-D64V was replaced by a synthetic PmlI-SphI DNA fragment encoding a modified MA in which one copy of PCP coding DNA (stop codons removed) replaced DNA coding for AA 44-132 of MA. The inserted DNA sequence is SEQ ID NO:73 and the corresponding amino acid sequence is SEQ ID NO:74. |
| 10 | pMDLg/pRRE-D64V-MA-PP7x2 | Third generation lentivirus packaging plasmid with two copies of PCP replacing AA 44-132 of MA. | The PmlI-SphI fragment of pMDLg/pRRE-D64V was replaced by a synthetic PmlI-SphI DNA fragment encoding a modified MA in which two copies of PCP coding DNA (stop codons removed) replaced DNA coding for AA 44-132 of MA. The inserted DNA sequence is SEQ ID NO:75 and the corresponding amino acid sequence is SEQ ID NO:76. |
| 11 | psPAX2-D64V-NC-MS2 | Second generation lentivirus packaging plasmid with MCP inserted after NC ZF2. | The SphI-SbfI fragment of psPAX2-D64V (without NC-MS2) was replaced by the SphI-SbfI fragment of pMDLg/pRRE-D64V-NC-MS2x1 (with one copy of MS2) to make psPAX2-D64V-NC-MS2. This is the second generation packaging plasmid with NC-MS2 insertion, corresponding to pMDLg/pRRE-D64V-NC-MS2x1 (Plasmid No. 4), which is the third generation packaging plasmid. |
| 12 | psPAX2-D64V-NC-PP7 | Second generation lentivirus packaging plasmid with one copy of PCP inserted after NC ZF2. | The SphI-SbfI fragment of psPAX2-D64V (without NC-PP7) was replaced by the SphI-SbfI fragment of pMDLg/pRRE-D64V-NC-PP7x1 (with one copy of PP7). |
| 13 | psPAX2-D64V-NC-MS2X2 | Second generation lentivirus packaging plasmid with 2 copies of MS2 inserted after NC ZF2 | The SphI-SbfI fragment of psPAX2-D64V (without NC-MS2x2) was replaced by the SphI-SbfI fragment of pMDLg/pRRE-D64V-NC-MS2X2 (with NC-MS2x2), so that now we added NC-MS2 to pspAX2-D64V. This is to make the second generation packaging plasmid with NC-MS2x2. |
| 14 | psPAX2-D64V-MA-MS2X2 | Second generation lentivirus packaging plasmid with two copies of MS2 replacing AA 44-132 of MA | The PvuI-SphI fragment of psPAX2-D64V was replaced by the Pvul-Sphl fragment of pMDLg/pRRE-D64V-MA-MS2X2. |
| **Mammalian Expression Plasmids** | | | |
| 15 | pSaCas9 | Adeno associated viral (AAV) plasmid expressing SaCas9 | pX601-AAV-CMV::NLS-SaCas9-NLS-3xHA-bGHpA;U6::BsaI-sgRNA (Addgene Cat. No. 61591) was cut with NotI and Acc65I (to remove the Sa sgRNA expression cassette) treated with DNA Pol I Klenow polymerase, and re-ligated by T4 DNA ligase. |
| 16 | pSaCas9^1xms2^ | Plasmid expressing SaCas9 mRNA with a MS2 stem loop at the 3’ untranslated region (UTR) | A synthetic dsDNA oligo was generated by annealing oligo 1xloop-F (SEQ ID NO:41) and oligo 1xloop-R (SEQ ID NO:42), which was then inserted by Infusion^TM^ reaction (Clontech) into the EcoRI site (after the stop codon) of pSaCas9. |
| 17 | pSaCas9^2xms2^ | Plasmid expressing SaCas9 mRNA with two MS2 stem loops at the 3’ UTR | A synthetic dsDNA oligo was generated by annealing oligo 2xloop-F (SEQ ID NO:43) and oligo 2xloop-R (SEQ ID NO:44), which was then inserted by Infusion^TM^ reaction (Clontech) into the EcoRI site of pSaCas9. |
| 18 | pSaCas9^3xms2^ | Plasmid expressing SaCas9 mRNA with three MS2 stem loops at the 3’ UTR | A synthetic dsDNA oligo was generated by annealing oligo 3xloop-F (SEQ ID NO:45) and oligo 3xloop-R (SEQ ID NO:46), which was then inserted by Infusion^TM^ reaction (Clontech)into the EcoRI site of pSaCas9-2xMS2. |
| 19 | pSaCas9^12xms2^ | Plasmid expressing SaCas9 mRNA with twelve MS2 stem loops at the 3’ UTR | The EcoR1-Xho1 fragment from pSL-MS2-12X (Addgene Cat. No. 27119), encoding the 12 MS2 stem loops, was inserted between the EcoR1-SalI sites of pSaCas9-1xMS2, which is after the stop codon of SaCas9 but before the polyA signal sequence. The original fragment coding for 1 MS2 stem loop was replaced with the 12 MS2 step loop fragment. |
| 20 | pSaCas9^1xPP7^ | Plasmid expressing SaCas9 mRNA with one PP7 stem loop after the stop codon of saCas9. | pSaCas9-^1xPP7^-HBB-sgRNA1^3’MS2^ was cut with Acc65I and NotI to excise the HBB sgRNA cassette. The plasmid was treated with DNA Pol I Klenow polymerase, and re-ligated with T4 DNA ligase. The starting plasmid had a PP7 aptamer coding sequence after the stop codon of the SaCas9 coding sequence and a MS2 aptamer coding sequence after the sgRNA coding sequence. The sgRNA cassette was removed to make a plasmid with a SaCas9 coding sequence followed by a PP7 aptamer coding sequence. |
| 21 | pSaCas9^12xpp7^ | Plasmid expressing SaCas9 mRNA with 12 PP7 stem loops at the 3’ UTR. | The BglII-BamHI fragment of pDZ617 pKAN 12xPP7 V4 (Addgene Cat. No. 72237), encoding the 12 PP7 loops, was inserted into the BamHI site of pSaCas9 (after the coding sequence of Sacas9 but before the HA tag, so that the HA tag is removed but the Sacas9 is complete). |
| 22 | pSaCas9-HBB-sgRNA1 | Plasmid expressing SaCas9 mRNA and HBB sgRNA1 targeting the region causing sickle cell disease. | A dsDNA oligo was generated by annealing oligo Sickle-g1F (SEQ ID NO:49) and oligo Sickle-g1R (SEQ ID NO:50), which was then inserted into the BsaI site of pX601-AAV-CMV::NLS-SaCas9-NLS-3xHA-bGHpA;U6::BsaI-sgRNA by T4 DNA ligase. |
| 23 | pSaCas9-HBB-sgRNA2 | Plasmid expressing SaCas9 mRNA and HBB sgRNA2 targeting the region causing sickle cell disease. | A synthetic DNA fragment was generated by annealing oligo Sickle-g2F (SEQ ID NO:47) and oligo Sickle-g2R (SEQ ID NO:48), which was then inserted into the BsaI site of pX601-AAV-CMV::NLS-SaCas9-NLS-3xHA-bGHpA;U6::BsaI-sgRNA by T4 DNA ligase. |
| 24 | pSaCas9-HBB-sgRNA1^3’ ms2^ | Plasmid expressing SaCas9 mRNA and the guide RNA for HBB; the 3’ of the sgRNA has a MS2 stem loop. | A synthetic DNA fragment (by Genscript) (SEQ ID NO:77) was cut with BsaI and NotI and inserted between the BsaI-NotI sites of pX601-AAV-CMV::NLS-SaCas9-NLS-3xHA-bGHpA;U6::BsaI-sgRNA. This DNA fragment encodes a HBB sgRNA1 with a MS2 aptamer at the 3’ end. |
| 29 | pSaCas9^1PP7^-HBB-sgRNA1^3’ ms2^ | Plasmid expressing SaCas9^1PP7^ mRNA and the guide RNA for HBB sgRNA^3’MS2^. | A single PP7 stem loop dsDNA oligo was generated by annealing synthetic DNA oligo PP7-F (SEQ ID NO:51) and oligo PP7-R (SEQ ID NO:52) and inserted into the EcoRI site of pSaCas9-HBB-sgRNA1-3’MS2 (after the stop codon of SaCas9 and before the PolyA signal sequence). |
| 30 | pSaCas9^1PP7^-HBB-sgRNA1^3’ PP7^ | Plasmid expressing SaCas9^1PP7^ mRNA and the guide RNA for HBB, the 3’ region of the sgRNA has a PP7 stem loop. | A synthetic DNA oligo (SEQ ID NO:81) was cut with KpnI and NotI and inserted between the KpnI-NotI sites of pSaCas9^1PP7^-HBB-sgRNA1^3’ MS2^. This results in excision of the U6-HBB sgRNA1-MS2 aptamer and replacing it with the U6-HBB sgRNA1-PP7 aptamer coding sequence. |
| 31 | pCK002-HBB-sgRNA1 | Lentiviral vector expressing SaCas9 and the sgRNA1 for HBB to treat sickle cell disease. | A dsDNA oligo was generated by annealing oligo sickle-g1-LV-F (SEQ ID NO:53) and oligo sickle-g1-LV-R (SEQ ID NO:54), which was then inserted into the BsmBI site of pCK002_U6-Sa-sgRNA(mod)_EFS-SaCas9-2A-Puro_WPRE (Addgene Cat. No. 85452). The dsDNA oligo is the guide sequence for HBB sgRNA1. |
| 32 | pAAV-HBB-sgRNA2 | Adeno associated viral (AAV) vector containing the HBB template for homologous recombination to correct the Sickle Cell Disease mutation causing sickle cell disease. The SaCas9 target sites were removed but the encoding amino acids were identical to the wild type. The vector also contains the cassette for U6 driven expression of sgRNA2, targeting the HBB gene close to the mutation causing sickle cell disease. | A synthetic DNA encoding the human HBB target template sequence and the U6 driven HBB sgRNA2 expression cassette (SEQ ID NO:82) was inserted into the NotI site of pAAV-MCS (Agilent Genomics, Cat. No. 240071). |
| 33 | pAAV-HBB-sgRNA1 | Adeno associated viral vector containing the HBB template for homologous recombination to correct the mutation causing sickle cell disease. The vector also contains the cassette for U6 driven expression of sgRNA1, targeting the HBB gene close to the mutation causing sickle cell disease. Since we found that sickle-sgRNA1 outperformed sickle-sgRNA2. | Primer Sickle-g1HD-F (SEQ ID NO:55) and Primer Sickle-g1HD-R (SEQ ID NO:56) were used to amplify the sickle-sgRNA1 expression cassette from pSaCas9-HBB-sgRNA1 using high fidelity DNA polymerase. The amplified DNA was inserted into the XhoI and XbaI sites of pAAV-HBB-sickle-sgRNA2 by Infusion (Clontech). |
| 34 | pAAV-HBB(n)-sgRNA1 | Adeno associated viral vector containing the HBB template for homologous recombination to change the wild type HBB gene into the version causing sickle cell disease. This will facilitate the detection of gene editing events in normal cells. The Sacas9 target sites were removed but the encoded amino acids were still wild type except for the disease causing mutation. The vector also contains the cassette for U6 driven expression of sgRNA1 for HBB, targeting the sequences close to the mutation causing sickle cell disease. | pAAV-HBB-sgRNA2 was cut with XhoI and XbaI, treated with Klenow DNA polymerase and the DNA was ligated to remove the cassette expressing sickle-sgRNA2. Then the NcoI-PasI fragment of the resulted plasmid was replaced by the annealed DNA from oligo HBB-tem-F (SEQ ID NO:57) and oligo HBB-tem-R (SEQ ID NO:58) to modify the template. Finally, the NcoI-BstxI of the plasmid was replaced by the NcoI-BstxI fragment from pAAV-HBB-sgRNA1 to add the HBB sgRNA1 expression cassette. |
| 35 | pFCK-HBB-sgRNA1 | Lentiviral vector containing the human HBB template (containing the mutation causing sickle cell disease) and the U6 driven HBB sgRNA1 expression cassette. | The sequences for human HBB template and the U6 driven HBB sgRNA1 expression cassette were amplified from pAAV-HBB(n)-sgRNA1 with primer HBB-LT-F (SEQ ID NO:57) and primer HBB-LT-R (SEQ ID NO:58) using high fidelity DNA polymerase (proofreading HotStart ReadyMix from KAPA Biosystems (Wilmington, MA)). The DNA was cut with XbaI and EcoRV and inserted into XbaI and EcoRV sites of FCK-ChR2-GFP (Addgene Cat. No. 15814). |
| 36 | pSaCas9^1xms2^-2x3’UTR | Plasmid having two copies 3’ untranslated region (UTR) from human *HBB* gene were inserted after the Sacas9 coding sequences and before the SM2 stem loop of pSaCas9^1xms2^. | A synthetic DNA of two copies of the human HBB 3’ UTR sequences (SEQ ID NO:83) was inserted between the BamHI-EcoRI sites of pSaCas9^1xms2^. The synthetic DNA was designed with a NheI site between the two human HBB 3’ UTR sequences. |
| 37 | pSaCas9^1xms2^-1x3’UTR | Plasmid having one copy of *HBB* gene 3’ untranslated region (UTR) was after the Sacas9 coding sequences and before the SM2 stem loop of pSaCas9^1xms2^. | pSaCas9^1xms2^-2x3’UTR was cut with NheI and EcoRI to remove one copy of HBB 3’ UTR. The backbone was treated with Klenow DNA polymerase and re-ligated with T4 DNA ligase. |
| 38 | pspCas9^1x ms2^ | Plasmid having one copy of *MS2* aptamer was added after the stop codon of sp. Cas9 mRNA. | A dsDNA oligo was generated by annealing oligo sp-loop1F (SEQ ID NO:88) and oligo sp-loop1R (SEQ ID NO:89) was inserted between the HindIII and EcoRI sites of pU6-sgRosa26-1_CBh-Cas9-T2A-BFP (Addgene Cat. No. 64216) by Infusion™ reaction (Takara, In-Fusion® HD Cloning Plus, Cat. 638909). |
| 39 | pLH-IL2RG-sp-sgRNA | A lentiviral expression plasmid expressing a spCas9 sgRNA targeting the start codon region of human IL2RG gene. | A dsDNA oligo was generated by annealing oligo Il2RG-sp-g1F1 (SEQ ID NO:90) and oligo Il2RG-sp-g1R (SEQ ID NO:91) was inserted into the BbsI site of pLH-sgRNA1 (Addgene Cat. No. 75388) by T4 DNA ligase. |
| 40 | pSaCas9^1xMS2^-HBB-sgRNA1 | A plasmid expressing SaCas9^1xMS2^ and *HBB* sgRNA1. | A synthetic dsDNA oligo was generated by annealing oligo 1xloop-F (SEQ ID NO:41) and oligo 1xloop-R (SEQ ID NO:42), which was then inserted by infusion into the EcoRI site of pSaCas9-HBB-sgRNA1. |
| 41 | pSaCas9^1xMS2^-2x3'UTR-HBB sgRNA^3’PP7^ 1x3'UTR | SaCas9 has a MS2 aptamer and two copies of HBB 3’ UTR, the HBB sgRNA1 has one copy of PP7 aptamer and one copy of HBB 3’ UTR. | A dsDNA oligo synthesized by Genscript (SEQ ID NO:84) was inserted into the Eag1 site of pSaCas91xMS2-2x3’UTR by T4 DNA ligase. The oligo contains in 5’ to 3’ order: a U6 promoter, a HBB sgRNA coding sequence (for Sickle mutation (g1)), a PP7 aptamer coding sequence, and a HBB 3’ UTR coding sequence. The U6 promoter direction is the same as the CMV promoter in the construct. |
| 42 | pSaCas9^1xMS2^-2x3'UTR-HBB sgRNA^3’PP7^ 2x3'UTR | SaCas9 has a MS2 aptamer and two copies of HBB 3’ UTR, the HBB sgRNA1 has one copy of PP7 aptamer and two copy of HBB 3’ UTR. | A dsDNA oligo synthesized by Genscript (SEQ ID NO:85) was inserted between the EcoRV and Not1 sites of pSaCas9^1xMS2^-2x3'UTR-HBB sgRNA^3’PP7^ 1x3'UTR (Plasmid No. 41). The sequence encoding one PP7 aptamer followed by one HBB 3’ UTR was replaced with a sequence encoding one PP7 aptamer followed by two HBB 3’ UTR sequences. |
| 43 | pSaCas9^1xms2^-2x3’UTR-HBB-sgRNA1^3’MS2^-2x3’UTR | One aptamer and 2 copies of HBB 3’ UTR sequences were added to both SaCas9 and HBB sgRNA1. | Synthetic oligo MS2-F1 (SEQ ID NO:92) and oligo MS2-R1 (SEQ ID NO:93) were annealed and the dsDNA oligo was inserted between the Afe1 and EvoRV sites of pSaCas9^1xMS2^-2x3'UTR-HBB sgRNA^3’PP7^ 2x3'UTR (Plasmid No. 42) by Infusion™ reaction (Takara, In-Fusion® HD Cloning Plus, Cat. 638909). |
| 44 | pLVX-ad-IL2RG-rep | Lentivirus vector to express the *sgRNA2* targeting IL2RG gene. It also contains the homologous recombination arms for the insertion of IL2RG cDNA into the target site. | pLVX-EF1α-IRES-zsGreen1 was digested with MluI and ClaI to remove the zsGreen1-expression cassette and ligated with an adaptor to introduce the NotI site. Then the synthetic NotI DNA fragment containing the IL2RG sgRNA2 expression cassette and the homologous recombination template were inserted into the NotI site of the modified vector (see SEQ ID 103). |
| 45 | pLVX-HBB-correct | Lentivirus vector to express the *HBB* *sgRNA1*. It also contains the homologous recombination arms for the correction of the Sickle mutation to the wild type *HBB* gene. | pLVX-EF1α-IRES-zsGreen1 was digested with MluI and ClaI to remove the zsGreen1-expression cassette and ligated with an adaptor to introduce the NotI site. Then the synthetic NotI DNA fragment (see SEQ ID 104) containing the HBB sgRNA1 expression cassette and the homologous recombination template (to change the Sickle mutation to the wild type HBB) were inserted into the NotI site of the modified vector. |
| 46 | pSpCas9-1loop-3’UTR | Plasmid for the expression of sp. Cas9 mRNA, with 2 copies of *HBB* 3’UTR and one copy of *MS2* aptamer after the *Cas9* stop codon. | pspCas9-1loop was cut with FseI+NotI to remove the sequence containing the 1x MS2 aptamer and the bGH polyA signal, and the vector backbone was recovered. Then the 600 bp FseI+Eag1 fragment from pX601-1loop-2x3'UTR (containing 2xHBB 3’UTR, 1xMS2 aptamer, and the bGH polyA signal) was ligated into the linearized the vector. |

**^a^Plasmid numbers are not continuous to keep the unique identity for each one in the lab.**
