# Supplementary material for: Delivering SaCas9 mRNA by lentivirus-like bionanoparticles for transient expression and efficient genome editing
Source: Nucleic Acids Res. 2019 Feb 13;47(8):e44. doi: 10.1093/nar/gkz093 (PMC6486560; doi:10.1093/nar/gkz093)
Supplement: Supplementary Data [file nar_47_8_e44_s1.zip › Supplementry_table_3_.docx]

**Supplementary Table 3**. Plasmid DNA used to transfect HEK293T cells to make lentiviral particles loaded with *SaCas9* mRNA^a^

|  | MCP-fused second generation packaging system | MCP-fused third generation packaging system | PCP-fused second generation packaging system | PCP-fused third generation packaging system | VPR or NEF mediated SaCas9 mRNA packaging |
| --- | --- | --- | --- | --- | --- |
| Gag-pol packaging plasmid (16.5μg) | pspAX2-D64V-NC-MS2 | pMDLg/pRRE-D64V-NC-MS2 | pspAX2-D64V-NC-PP7 | pMDLg/pRRE-D64V-NC-PP7 | pspAX2-D64V |
| *SaCas9* plasmid (12μg) | pSaCas9^1x-MS2^ | pSaCas9^1x-MS2^ | pSaCas9^1xPP7^ | pSaCas9^1xPP7^ | pSaCas9^1x-MS2^ |
| pMD2G (μg) | 8 | 8 | 8 | 8 | 8 |
| pRSV-REV (μg) | 0 | 4.5 | 0 | 4.5 | 0 |
| VPR or NEF MCP fusion plasmid (μg) | 0 | 0 | 0 | 0 | 12 |

^a^13x10^6^ cells were seeded in 15-cm dishes 24 hours before transfection.
